# Effects of maternal allergy and supplementation with ω‐3 fatty acid and probiotic on human milk oligosaccharides

**DOI:** 10.1111/pai.70162

**Published:** 2025-08-01

**Authors:** Ahmed Al‐Kaabawi, Eva Landberg, Magalí Martí, Elisabet Severin, Lina Tingö, Karel Duchén, Maria C. Jenmalm

**Affiliations:** ^1^ Department of Biomedical and Clinical Sciences, Division of Inflammation and Infection Linköping University Linköping Sweden; ^2^ Department of Clinical Chemistry, and Department of Biomedical and Clinical Sciences Linköping University Linköping Sweden; ^3^ Department of Biomedical and Clinical Sciences, Division of Children's and Women's Health Linköping University Linköping Sweden; ^4^ Allergy Center University Hospital Linköping Sweden

**Keywords:** clinical trial, human milk, human milk oligosaccharides, *Limosilactobacillus reuteri*, maternal allergy, secretory immunoglobulin a, ω‐3 polyunsaturated fatty acids

## Abstract

**Background:**

Human milk oligosaccharides (HMOs) are complex carbohydrates that act as prebiotics, supporting infants' gut microbial colonization and immune development. HMO levels are influenced by several maternal factors, including genetics, diet, and health status. In this study, we aim to investigate the effects of ω‐3 PUFA (polyunsaturated fatty acids) and *Limosilactobacillus* (*L*.) *reuteri* supplementation on HMO levels in colostrum and mature milk. Another aim is to compare HMO levels between allergic and non‐allergic mothers and to explore the correlation between HMOs and secretory immunoglobulin A (SIgA) in milk.

**Methods:**

Milk samples (*n* = 136) were collected from mothers enrolled in a clinical trial (PROOM‐3) designed to investigate the effect of pre‐ and postnatal supplementation with ω‐3 PUFA and *L. reuteri* on allergy development in early childhood. HMOs were measured in colostrum and mature milk collected 3 months postpartum using high‐performance anion exchange chromatography. SIgA was measured in colostrum, 1‐, 2‐, 3‐, and 4‐month milk using ELISA.

**Results:**

The supplements did not affect HMO levels in colostrum or mature milk. However, maternal supplementation with ω‐3 PUFA decreased the HMO diversity over time. Additionally, allergic mothers expressed significantly lower levels of several HMOs compared to non‐allergic mothers. Additionally, SIgA correlated positively with fucosylated and negatively with sialylated HMOs.

**Conclusion:**

Supplementation with ω‐3 PUFA could reduce the HMO diversity over the course of lactation. Also, maternal allergy seems to be associated with a reduction in levels of several HMOs. Furthermore, there is a possible dynamic interplay between HMOs and SIgA in milk.

**Trial Registration:**

ClinicalTrials.gov‐ID: NCT01542970

**Key message**

Human milk oligosaccharides (HMOs) can affect infant health and development. This study investigated the association of several maternal factors with HMO levels. We showed that maternal supplementation with ω‐3 PUFA reduced HMO diversity over time, which might benefit infant growth. We also observed that maternal allergy is associated with lower levels of several HMOs. Finally, we found several correlations between HMOs and SIgA levels in milk, which might hint at a complex interaction between the two mediators.

## 1 INTRODUCTION

Human milk is a unique fluid tailored to meet the nutritional needs of infants.^1^ Beyond its nutritional role, it also protects against pathogens through antimicrobial peptides, supports the development of the infant's immune system by fostering the gut microbiota, and strengthens intestinal barrier function.^1^, ^2^ These immunomodulatory effects are orchestrated through several bioactive molecules, such as immunoglobulins, extracellular vesicles, and human milk oligosaccharides.^2^ Human milk oligosaccharides (HMOs) are complex carbohydrates produced by the epithelial mammary gland and constitute the third most common solid component in milk, after lipids and lactose.^3^ These oligosaccharides are largely indigestible, and the majority act as prebiotics, nourishing the neonate's intestinal microbiota.^4^

Over 200 structurally distinct HMOs have been identified, varying in size from 3 to 32 monosaccharides. These monosaccharides are glucose, galactose, N‐acetylglucosamine, fucose, and sialic acid, primarily in the form of N‐acetylneuraminic acid.^5^, ^6^ Each HMO consists of a lactose core and an oligosaccharide chain, which can be neutral or undergo fucosylation and/or sialylation, resulting in fucosylated and sialylated HMOs.^3^, ^6^ The HMO levels and availability are strongly influenced by two genes, Secretor (Se) and Lewis (Le), which encode the α1‐2‐fucosyltransferase (FUT2) and α1‐3/4‐fucosyltransferase (FUT3) enzymes, respectively. Mutations in these genes disrupt fucosylation, reducing or eliminating specific HMOs. Variations in Se and Le gene functionality result in four distinctive milk phenotypes: Secretor Lewis‐positive (Se^+^Le^+^), non‐Secretor Lewis‐positive (Se^−^Le^+^), Secretor Lewis‐negative (Se^+^Le^−^), and non‐Secretor Lewis‐negative (Se^−^Le^−^).^4^, ^5^, ^6^ The distribution of these four phenotypes varies geographically.^7^, ^8^ Among European women, approximately 71% are Se+Le+, 18% are Se‐Le+, 7% are Se+Le‐, and 4% are Se‐Le‐.^7^

Previous studies investigated the role of HMOs in infant health and immunity. HMOs attenuated chemokine release from human intestinal epithelial cells, potentially regulating gut immunity.^9^ Moreover, HMO supplementation enhanced the intestinal barrier function by reducing gut permeability in rats.^10^ In a murine model, HMO supplementation alleviated food allergy symptoms, along with an increase in intestinal T regulatory cells (Treg) and a decrease in mast cells.^11^ In humans, the HMO profile plays a fundamental role in shaping the infant gut microbiota by selectively promoting the growth of specific bacteria, such as *Bifidobacterium infantis*, *B. breve*, and *B. bifidum*.^4^ HMO levels have been linked to a reduced risk of cow's milk allergy and atopic dermatitis in infants.^12^ However, HMOs not only shape the infant's gut microbiota but also appear to influence the mother's milk microbiota.^13^, ^14^, ^15^ This milk microbial modification may affect the mammary epithelium and modulate the levels of other immune mediators in milk, such as Immunoglobulin A (IgA), either directly or via their bacterial metabolites, like short‐chain fatty acids (SCFAs).^16^

Allergies are increasing in affluent societies,^17^ potentially due to dietary shifts, such as an increased ω‐6/ω‐3 ratio,^18^ and reduced exposure to environmental microbes.^19^ Pre‐ and postnatal supplementation with ω‐3 polyunsaturated fatty acids (PUFA)^20^ and probiotics^21^ appears to reduce the risk of allergy in infants. While the mechanisms remain unclear, breastfeeding could play a mediating role. Interestingly, a previous trial found that maternal supplementation with probiotics altered several HMO levels.^22^ Another clinical trial showed that maternal supplementation with *Limosilactobacillus* (*L*) *reuteri* and sialic acid increased certain HMO levels through the gut‐breast axis.^23^ Maternal diet could also influence the HMO profile. It was previously shown that fat and carbohydrate intake altered HMO composition.^24^ Moreover, food items such as fish, fiber, fruits, and unsaturated fatty acids have been linked to changes in HMO levels.^25^, ^26^

In this study, we investigated the effect of pre‐ and postnatal ω‐3 PUFA and *L*. *reuteri* supplementation on HMO levels in colostrum and mature (3 months) milk. We also explored the effect of maternal allergy on the HMO profile. Additionally, we determined the association between HMO and SIgA concentrations in milk at different time points.

## 2 METHODS

### 2.1 Study population

This study is part of an ongoing allergy prevention, double‐blind, randomized, placebo‐controlled, multicentric trial (ClinicalTrials.gov‐ID: NCT01542970). Families with a history of allergies were invited to take part in the trial. Additional inclusion criteria were the mother's intention to breastfeed for the first 3 months, absence of any known fish allergy, and avoidance of other supplement use. Allergic disease was defined as atopic dermatitis, food allergy, allergic rhinoconjunctivitis, or asthma, based on self‐reported data, and the diagnosis was then verified by a clinician based on the reported symptoms. The trial primarily aims at investigating the effects of pre‐ and postnatal supplementation with *L*. *reuteri* and ω‐3 PUFA on allergy development in the first 2 years of life. Randomization was carried out based on a computer‐generated list created using blocked randomization with block sizes of eight, prepared by an independent statistician who was not otherwise involved in the study. Women were randomized into one of four supplementation groups receiving either: (1) ω‐3 PUFA + *L. reuteri* (OL), (2) ω‐3 PUFA + Placebo (OP), (3) Placebo + *L. reuteri* (PL), and (4) Placebo + Placebo (PP). Pregnant women took 10 drops of *L. reuteri* DSM 17938 (BioGaia AB, Stockholm, Sweden) oil twice daily from gestational week 20 until delivery, equivalent to 10^9^ colony‐forming units (CFUs) per serving. After delivery, the child is given 5 drops once daily, equivalent to 10^8^ CFUs per serving during the first year of life; the corresponding placebo contains refined coconut and peanut oil without *L. reuteri*. The ω‐3 PUFA supplement consists of three 1000 mg Pikasol capsules (Orkla Health, Lund, Sweden); each capsule containing 640 mg ω‐3 PUFAs, of which 35% is eicosapentaenoic acid (EPA) and 25% is docosahexaenoic acid (DHA), and taken twice daily by the pregnant women from gestational week 20 until 3 months postpartum, while the corresponding placebo consists of olive oil. The infants received the ω‐3 PUFA indirectly through breastfeeding till 3 months of age. This study was approved by the Regional Ethics Committee in Linköping (Dnr 2011/45–31), and written informed consent was obtained from all participants.

### 2.2 Breast milk collection and experimental procedures

In this substudy, all mothers (*n* = 136) who had donated both colostrum and 3‐month milk samples prior to the start of the study were included. Breast milk samples were collected 1–3 days postpartum (colostrum), and then monthly up to 4 months. To standardize sampling, mothers were instructed to collect milk immediately after the first morning feeding, 2 min after starting breastfeeding, by a sterile manual pump (ArtaPlast, Tyresö, Sweden). Milk samples were stored in the participants' home freezers and, within a few weeks, delivered frozen to the laboratory, where they were stored at −70°C until analysis. High‐performance anion‐exchange chromatography with pulsed amperometric detection (HPAEC‐PAD) was used to measure 14 major HMOs in colostrum and mature (3 months) milk samples (Table 1). HMO types, including fucosylated, neutral, and sialylated, as well as total HMOs, were calculated as the sum of the corresponding measured HMOs (Table 1). In this paper, Secretor Lewis positive (Se^+^Le^+^) is referred to as Se, while non‐Secretor Lewis positive (Se^−^Le^+^) as nSe. Inter‐batch variability, based on an nSe 3‐month milk control, showed a mean coefficient of variation (C.V.%) of 20.2% for the 10 detectable HMOs, with no individual HMO exceeding 30% (Table S2). Enzyme‐Linked Immunosorbent Assay (ELISA) was used to measure the Secretory IgA (SIgA) levels in colostrum, 1‐, 2‐, 3‐, and 4‐month milk samples. More detailed information on experimental procedures is provided in Appendix S1.

**TABLE 1:** Names, abbreviations, types, and secretion status of the 14 analyzed HMOs in colostrum and mature human milk samples.

Name	Abbreviation	Types	Secreted by
2’‐Fucosyllactose	2’‐FL	Fucosylated	Se+
3‐Fucosyllactose	3‐FL	Fucosylated	All
Lactodifucotetraose	LDFT	Fucosylated	Se+
Lacto‐N‐fucopentaose I	LNFP I	Fucosylated	Se+
Lacto‐N‐fucopentaose II	LNFP II	Fucosylated	Le+
Lacto‐N‐fucopentaose III	LNFP III	Fucosylated	All
Lacto‐N‐difucohexaose I	LNDFH I	Fucosylated	Se + Le+
Lacto‐N‐tetraose	LNT	Neutral	All
Lacto‐N‐neotetraose	LNnT	Neutral	All
3′‐sialyllactose	3’‐SL	Sialylated	All
6′‐sialyllactose	6’‐SL	Sialylated	All
Sialyl‐lacto‐N‐tetraose b	LST b	Sialylated	All
Sialyl‐lacto‐N‐neotetraose c	LST c	Sialylated	All
Disialyl‐lacto‐N‐tetraose	DSLNT	Sialylated	All

### 2.3 Statistical analyses

Data analyses comparing the four supplementation groups were conducted by an unblinded researcher (author initials M.M.) using R (version 4.4.2). Normality was assessed using the Shapiro–Wilks test using R. Maternal demographic characteristics and milk phenotypes across supplementation groups were analyzed using the Chi‐square test (or Fisher–Freeman–Halton Exact Test when any cell count is less than 5) for ordinal variables and the Kruskal–Wallis test for continuous variables. Also, maternal demographic characteristics and milk phenotypes across maternal allergy status were analyzed using the Chi‐square test (or Fisher's Exact Test when any cell count is less than 5) for ordinal variables and the Mann–Whitney *U* test for continuous variables. HMO alpha diversity was calculated with the Shannon diversity index, using the *vegan* package (Oksanen et al., 2025) in R. Changes in HMO levels and diversity over time were assessed using the Wilcoxon matched‐pairs signed‐rank test in GraphPad Prism (Version 10.0.2, CA, USA). The Aligned Rank Transform (ART) ANOVA was used to test any interaction effect between maternal allergy and HMO diversity change in each supplementation group using the ARTool package (Kay et al., 2025) in R. Differences in HMO levels and diversity between the supplementation groups were examined with the Kruskal–Wallis test for independent samples, followed by Benjamini and Hochberg's false discovery rate (FDR) correction for multiple comparisons. To compare HMO levels between allergic and non‐allergic mothers, the Mann–Whitney *U* test for independent samples was performed using IBM SPSS Statistics (version 29.0.2, NY, USA), with Benjamini‐Hochberg's FDR correction at 5% applied separately for the 14 measured HMOs, the four calculated HMO types (fucosylated, neutral, sialylated, and total HMOs), and HMO diversity using GraphPad Prism. The two‐way permutational multivariate analysis of variance (PERMANOVA) was used to test any interaction effect between maternal allergy status and supplementation group using the vegan package in R. Correlations between HMOs and SIgA were assessed with Spearman's rho (r_s_) nonparametric test using IBM SPSS. All figures were created with GraphPad Prism, except Figure S1 was generated in Microsoft Excel (Version 16.0).

## 3 RESULTS

### 3.1 Description of the study population

Maternal demographic characteristics and milk phenotypes were compared across the supplementation groups (Table 2) and maternal allergy status (Table S3). Maternal allergy cases were significantly lower in the OP group compared to the other supplementation groups (*p* = .014) (Table 2). No other significant differences were observed in maternal demographic characteristics and milk phenotypes across the supplementation groups or the maternal allergy status (Table 2, Table S3).

**TABLE 2:** Distribution of the mothers' demographic characteristics and milk phenotypes between the four supplementation groups.

	OL	OP	PL	PP	Total
Characteristics					
Number of participants	36 (26.5%)	30 (22%)	36 (26.5%)	34 (25%)	136 (100%)
Age (years)	30 (4.2)	30.5 (4.4)	32 (4.1)	31 (4.2)	31 (4.2)
Start of supplementation (weeks)	20 (1.5)	20 (1.6)	20 (2.2)	20 (1.4)	20 (1.7)
Time of delivery (weeks)	40 (1.3)	40 (1.3)	40 (2.2)	40 (1.4)	40 (1.6)
Mode of delivery (Caesarean)	2 (5.6%)	6 (20%)	2 (5.6%)	1 (3%)	11 (8.1%)
Baby's sex (male)	11 (30.6%)	13 (43.3%)	19 (52.8%)	18 (54.5%)	61 (44.9%)
Exclusive breastfeeding at 3 months	22 (68.8%)	24 (82.8%)	25 (80.6%)	23 (74.2%)	94 (69.1%)
Maternal allergy (allergic)	26 (72.2%)	11 (36.6%)	22 (61.1%)	24 (70.6%)	83 (61%)
Milk phenotypes					
Se^+^Le^+^	25 (69.4%)	17 (56.7%)	23 (63.9%)	30 (88.2%)	95 (70%)
Se^−^Le^+^	7 (19.4%)	7 (23.3%)	9 (25%)	2 (5.9%)	25 (18%)
Se^+^Le^−^	3 (8.3%)	5 (16.7%)	3 (8.3%)	2 (5.9%)	13 (10%)
Se^−^Le^−^	1 (2.8%)	1 (3.3%)	1 (2.8%)	0 (0%)	3 (2%)

*Note*: The median and standard deviation were shown for age, start of supplementation, and time of delivery, while counts and percentages are shown for the other parameters. Maternal allergy had a significantly lower count in the OP group (*p* = .014). No other significant differences in the number of participants, characteristics, or milk phenotypes were observed between the supplementation groups. Missing demographic data is described in Table S4. Supplementation groups: OL (ω‐3 PUFA + *L. reuteri*), OP (ω‐3 PUFA + Placebo), PL (Placebo + *L. reuteri*), and PP (Placebo + Placebo).

### 3.2 HMO levels in colostrum and mature milk

In secretors' (Se) milk, the predominant HMO was 2'‐FL in colostrum and mature milk. In non‐secretors' (nSe) milk, LNT was the highest HMO in colostrum, replaced by 3‐FL in mature milk. In the whole cohort, 3‐FL was the only HMO to increase over time, tripling its concentration in mature milk. LDFT, LNFP II, and LST b levels remained stable over time, while all other HMOs declined significantly. Fucosylated HMOs were the predominant HMO type, followed by the neutral HMOs and sialylated HMOs in both colostrum and mature milk. Overall, the total HMO concentration decreased over time, from colostrum to mature milk (Table 3, Figure S1). The HMO diversity was higher in Se than nSe milk samples at both time points (Figure S2).

**TABLE 3:** Median concentration (g/L), interquartile range (IQR), and percentage of HMOs in colostrum and mature (3 months) milk samples.

HMOs	Median (IQR) in colostrum	% in colostrum	Median (IQR) in mature milk	% in mature milk
2’‐FL	4.41 (1.98)	26.09	2.43 (1.60)	26.22
3‐FL	0.45 (0.52)	2.66	1.52 (1.01)	16.34
LDFT	0.34 (0.35)	2.01	0.34 (0.24)	3.66
LNFP I	2.66 (1.65)	15.76	0.46 (0.67)	4.91
LNFP II	0.42 (0.50)	2.49	0.43 (0.42)	4.60
LNFP III	0.60 (0.39)	3.58	0.49 (0.27)	5.31
LNDFH I	1.75 (0.77)	10.39	0.87 (0.59)	9.34
LNT	2.12 (1.55)	12.58	1.24 (0.79)	13.33
LNnT	1.74 (0.48)	10.31	0.78 (0.38)	8.45
3’‐SL	0.18 (0.10)	1.07	0.11 (0.05)	1.15
6’‐SL	0.76 (0.35)	4.46	0.14 (0.11)	1.52
LST b	0.06 (0.06)	0.34	0.06 (0.05)	0.64
LST c	0.46 (0.31)	2.70	0.03 (0.03)	0.29
DSLNT	0.94 (0.60)	5.57	0.39 (0.24)	4.23
Fucosylated HMOs	9.55 (4.70)	60.21	6.23 (2.11)	69.53
Neutral HMOs	3.83 (1.90)	24.25	1.98 (1.22)	22.07
Sialylated HMOs	2.47 (1.10)	15.54	0.75 (0.40)	8.40
Total HMOs	15.85 (4.66)	100	9.16 (2.61)	100

### 3.3 Effect of ω‐3 PUFA and *L. reuteri* supplementation on HMO levels and diversity

**FIGURE 1:**
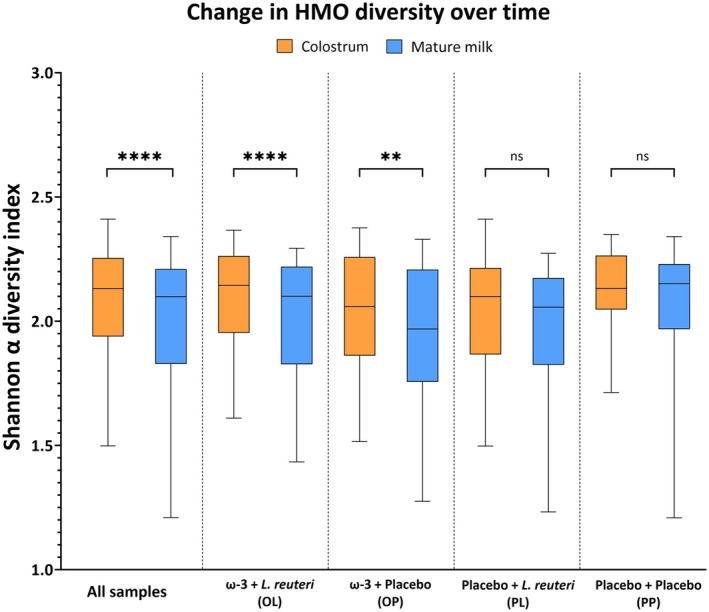
Boxplots with median and whiskers extending from minimum to maximum demonstrate the change in HMO diversity over time between colostrum and mature milk samples. The Wilcoxon matched‐pairs signed rank test showed a significant decline in total HMO diversity over time in the entire study population. Groupwise, mothers supplemented with ω‐3 PUFA, OL, and OP groups showed a significant decrease in HMO diversity over time. However, no such effect was observed in the PL or PP groups. *p* < .001 = **, *p* < .0001 = ****, ns = no significance.

We found no significant differences in HMO levels or diversity between the four supplementation groups in colostrum or mature milk. However, the HMO diversity declined significantly over time, from colostrum to mature milk, in the whole study population (*p* < .0001). When investigating the change in HMO diversity over time in each of the supplementation groups, using the Wilcoxon matched‐pairs signed‐rank test, we observed a decrease in the OL group (*p* < .0001) and the OP group (*p* = .0093), both of which received ω‐3 PUFA supplementation, while no significant changes were observed in the PL and the PP groups (Figure 1). These findings suggest that ω‐3 PUFA supplementation might reduce the HMO diversity over the first 3 months of lactation.

Using a multivariate analysis, we examined any potential interaction between the change in diversity over time and maternal allergy status. The Aligned Rank Transform (ART) ANOVA, conducted separately within each supplementation, found no significant interaction between maternal allergy and diversity change over time (Table S3).

### 3.4 Effect of maternal allergy on HMO levels

**FIGURE 2:**
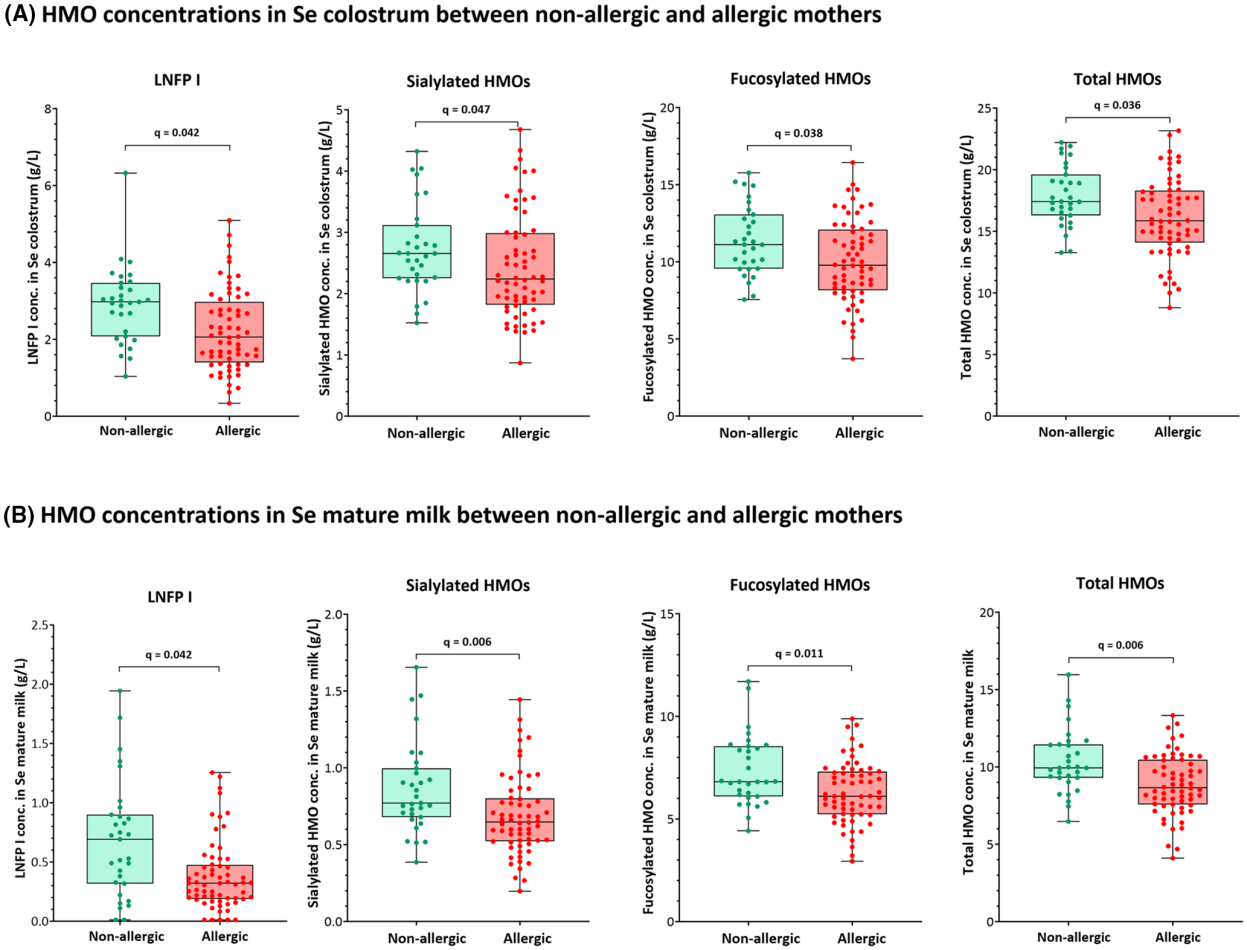
Boxplots with median and whiskers extending from minimum to maximum show the difference in HMO concentrations between non‐allergic and allergic Se mothers in colostrum and mature milk. LNFP I, sialylated, fucosylated, and total HMO concentrations were significantly higher in non‐allergic mothers (*n* = 31) than in allergic ones (*n* = 64) at both time points. The adjusted *p*‐value (q) was calculated using the Kruskal–Wallis test, followed by Benjamini‐Hochberg's correction at a 5% FDR. A *q*‐value below 0.05 was considered statistically significant.

We explored whether maternal allergy status (allergic versus non‐allergic) is associated with changes in HMO levels and diversity in colostrum and mature milk. In mature milk, non‐allergic mothers had significantly higher 2'‐FL, LNFP I, LNT, 3'‐SL, 6'‐SL, LST b, DSLNT, sialylated, neutral, and total HMO concentrations compared to allergic mothers (Table S6, Figure S3). In the Se subpopulation (Se^+^Le^+^), the differences in HMO levels were consistent at both time points. Se non‐allergic mothers had significantly higher LNFP I, sialylated, fucosylated, and total HMOs than allergic mothers in colostrum and mature milk (Table S6, Figure 2). This data suggests that maternal allergy might be associated with reduced levels of several HMOs.

We also investigated any potential interaction effect between the maternal allergy status and supplementation groups using the two‐way permutational multivariate analysis of variance (PERMANOVA). We found no significant interaction between maternal allergy status and supplementation groups in either colostrum or mature milk (Table S7).

### 3.5 Association between HMOs and SIgA in human milk

We also explored whether HMO levels are linked to SIgA levels in milk. Using the Spearman rank‐order (r_s_) test, we identified several significant correlations, with weak to moderate strength, between HMOs and SIgA at different time points. The strongest correlations were observed at the corresponding time points: SIgA and HMOs in colostrum, and SIgA and HMOs in 3‐month milk (Figure S4). SIgA exhibited a positive correlation with fucosylated and consequently total HMOs, a weaker negative correlation with sialylated HMOs, and no correlation with neutral HMOs (Table S8, Figure S4).

**FIGURE 3:**
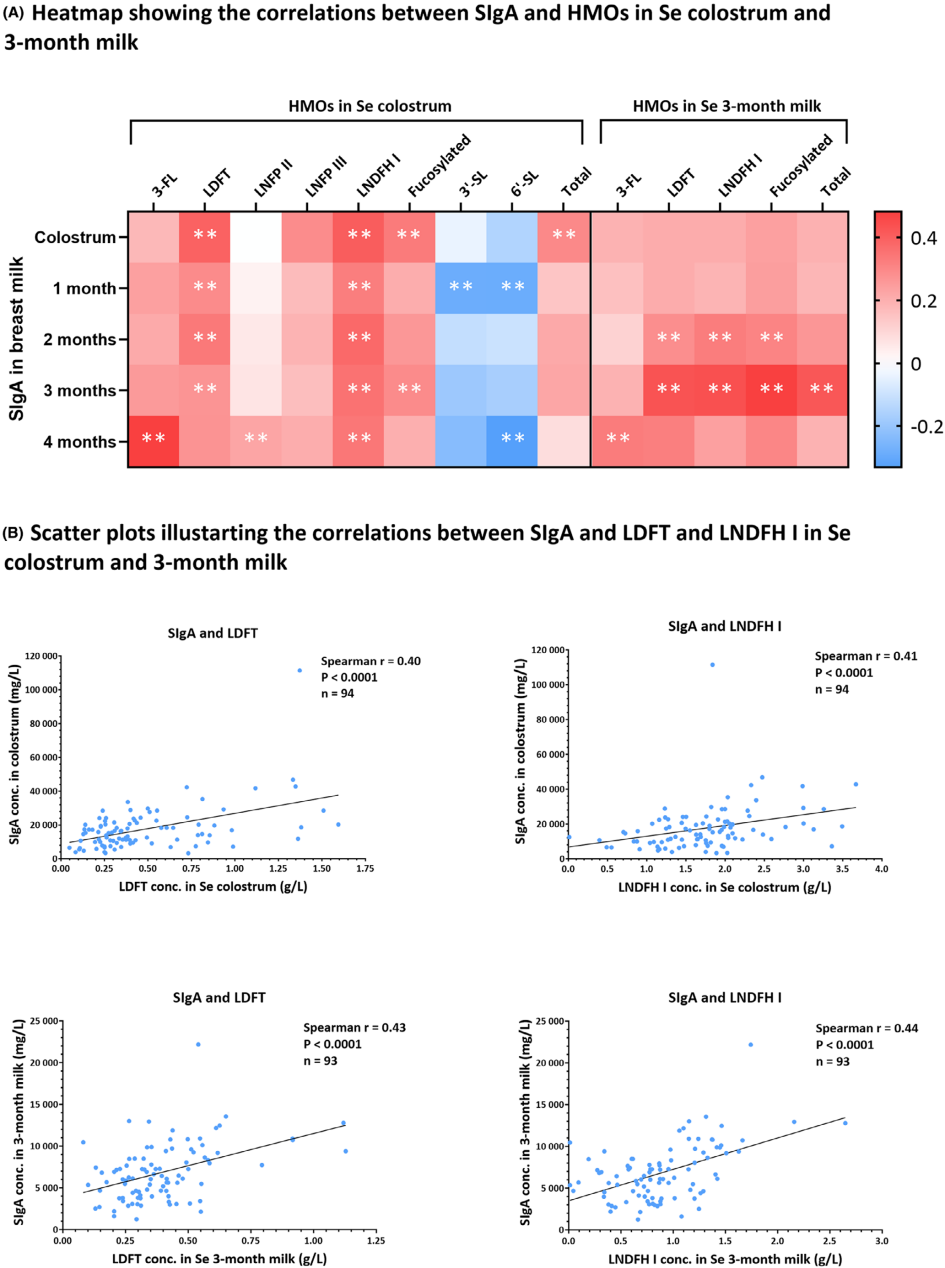
Graphs showing the correlation between SIgA and HMOs in Se colostrum and 3‐month milk. (A) Heatmap showing the significant associations, with weak to moderate strength, between SIgA in milk at different time points and HMOs in Se colostrum and 3‐month milk. ** corresponds to *p* < .001. (B) Scatter plots illustrating the association between SIgA and two HMOs, LDFT and LNDFH I, in corresponding Se colostrum and mature milk collected at 3 months postpartum. In Se colostrum (*n* = 94), the correlation was significant (*p* < .0001) with Spearman's rho (*r*_s_) of 0.40 for LDFT and 0.41 for LNDFH I with SIgA. In Se 3‐month milk (*n* = 93), the correlation was significant (*p* < .0001) with *r*_s_ of 0.43 for LDFT and 0.44 for LNDFH I with SIgA. A simple linear regression line (solid line) is fitted through the data.

In the Se subpopulation, similar and more pronounced associations between SIgA and HMOs were detected, especially with LDFT and LNDFH I (Figure 3). In Se colostrum (*n* = 94), SIgA correlated positively with both LDFT (*r*_s_ = 0.40, *p* < .0001) and LNDFH I (*r*_s_ = 0.41, *p* < .0001). Similarly, in Se 3‐month milk (*n* = 93), SIgA correlated directly with both LDFT (*r*_s_ = 0.43, *p* < .0001) and LNDFH I (*r*_s_ = 0.44, *p* < .0001) (Figure 3). Likewise, SIgA showed a positive correlation with fucosylated and total HMOs in Se colostrum and 3‐month milk (Figure S5). In the nSe (Se^−^Le^+^) subpopulation, SIgA showed a stronger correlation with LNFP III in nSe colostrum (*r*_s_ = 0.58, *p* = .003) and with LNFP II in nSe 3‐month milk (*r*_s_ = 0.62, *p* = .001) (Table S8). These weak to moderate correlations might indicate an interplay between HMOs and SIgA in milk.

## 4 DISCUSSION

This study examined three key outcomes: the effect of *L. reuteri* and ω‐3 PUFA on HMO concentrations and diversity, the impact of maternal allergy on HMO levels, and the relationship between HMOs and SIgA in milk. We discovered that pre‐ and postnatal supplementation with ω‐3 PUFA is associated with a decrease in HMO diversity over time, between colostrum and mature milk. Also, allergic mothers expressed lower levels of 2'‐FL, LNFP I, LNT, 3'‐SL, 6'‐SL, LST b, DSLNT, sialylated, neutral, and total HMOs than non‐allergic mothers in mature milk. In the Se subpopulation, allergic mothers had lower levels of LNFP I, fucosylated, sialylated, and total HMOs than non‐allergic mothers in both colostrum and mature milk. In addition, SIgA in milk showed weak to moderate positive correlations with fucosylated HMOs and weak inverse correlations with sialylated HMOs.

The mother‐infant dyad is established through the placenta during pregnancy and continues via breastfeeding postpartum.^19^, ^27^ This physiological connection, facilitated by the exchange of fluids, may underlie one of the mechanisms through which maternal health influences infant health. In this study, we reported the levels of the 14 most highly expressed HMOs, and despite the presence of over 200, the quantity of the major 14 to 16 HMOs can account for 75%–90% of the total.^4^, ^28^ Recent reviews reported that the median HMO concentration in colostrum is approximately 16.7 g/L and decreases to around 10.3 g/L in mature milk.^4^, ^29^, ^30^ We reported a slightly lower median HMO concentration, which is probably because other studies included more HMOs. However, the 14 HMOs we measured in this study were in the range of what was previously reported, indicating the accuracy of our results. Furthermore, our study cohort had a similar milk phenotype distribution (70% Se+Le+, 18% Se‐Le+, 10% Se+Le‐, and 2% Se‐Le‐) to that reported among European women (71% Se+Le+, 18% Se‐Le+, 7% Se+Le‐, and 4% Se‐Le‐).^8^

The HMO diversity differs between women from different geographical locations.^8^ However, it tends to remain stable over the course of lactation.^31^, ^32^ Surprisingly, our analysis revealed a significant decline in HMO diversity over time in the entire cohort. Upon closer examination of the supplementation effects on HMO diversity, we found that the two supplementation groups receiving ω‐3 PUFA displayed a significant reduction in diversity over time, while no difference was observed in the two other groups not receiving ω‐3 PUFA. These findings suggest that ω‐3 PUFA supplementation may have contributed to the observed reduction in HMO diversity in our study. Interestingly, this reduction could be beneficial for infant health, as previous research showed an inverse correlation between HMO diversity and infant growth.^32^, ^33^ On the other hand, probiotic supplementation did not affect HMO levels or diversity, which might be because *L. reuteri* supplementation was discontinued directly after delivery, whereas ω‐3 PUFA intake continued until 3 months postpartum, perhaps allowing a more pronounced effect on the HMO composition.

Maternal diseases with inflammatory manifestations, such as obesity and gestational diabetes mellitus (GDM), may impact the HMO concentrations.^34^ It was previously reported that mothers with GDM had significantly lower fucosylated HMOs in colostrum compared to those with normal glucose tolerance. Interestingly, the fucosylated HMO levels increased gradually from Se colostrum to transition milk in GDM, suggesting a postpartum normalization of HMO composition as GDM resolved.^35^ In this study, we found that allergic mothers had significantly lower levels of HMOs, including several fucosylated and sialylated ones, than non‐allergic mothers. The difference was more evident in mature milk compared to colostrum. This may be due to the strong immunomodulatory effects of pregnancy, which promote immunotolerance and might temporarily mask preexisting immune differences between allergic and non‐allergic mothers.^36^ As the postpartum period progresses and the influence of pregnancy on the immune system diminishes, the impact of maternal allergy on HMO composition may become more apparent. Several studies have compared milk composition between allergic versus non‐allergic mothers, revealing notable differences. Allergic mothers showed higher cytokine and chemokine levels, including interleukin (IL)‐4, IL‐5, IL‐6, CXCL8, and IL‐13, along with lower ovalbumin‐specific IgA concentrations.^37^, ^38^ Additionally, proteomic analysis identified significant variations in milk proteins, with allergic mothers exhibiting elevated levels of protease inhibitors and lipoproteins.^39^ Interestingly, a previous study examining SCFA levels, which are bacterial metabolites of HMOs, found that allergic mothers also had lower SCFA concentrations in their milk,^40^ possibly due to a lower HMO availability, as we show in this paper.

Furthermore, we assessed the correlation between HMOs and SIgA in milk. We found weak to moderate positive correlations between SIgA and fucosylated HMOs and weak negative correlations with sialylated HMOs. Previous studies failed to find such correlations between HMOs and SIgA.^41^, ^42^ However, these studies included 6‐month milk compared to colostrum and 3‐month milk in our study. Also, we used HPAEC‐PAD, which is a quantitative method with good repeatability results,^43^ while they used mass spectrometry, which only gives relative intensities and is generally not recommended for quantitative purposes.^44^ The correlations we found in this paper between HMOs and SIgA might be attributed to a complex interaction within the mammary gland involving several immune modulators, such as HMOs, SCFAs, SIgA, and microbiota.^13^, ^14^, ^15^, ^16^, ^45^ Speculatively, a higher HMO composition may initiate a regulatory cascade, starting with the creation of a more favorable environment for residing bacteria in the mammary gland, thereby increasing SCFA production and other postbiotics,^3^ which might in turn stimulate SIgA secretion in milk. The correlation might also be mediated through the entero‐mammary pathway, also known as the gut‐breast axis. Previous literature suggested a possible translocation of maternal gut bacteria to the lactating mammary gland, which could in turn influence milk components.^46^ Further, maternal gut microbiota composition may impact IgA‐producing plasma cells in the intestine, which then migrate to the mammary gland during late pregnancy and lactation, contributing to IgA production in milk.^47^, ^48^ Another important factor in this complex interaction is the FUT2 genotype. A transcriptome‐wide association study identified FUT2 as a key gene influencing gut microbiota composition.^49^ In addition, the mother's secretor status (FUT2 genotype) influenced the gut microbiota composition changes during pregnancy, resulting in distinct microbiota profiles between secretors and non‐secretors.^50^ The previous findings suggest that FUT2‐mediated fucosylation influences the HMO profile and the maternal gut microbiota composition, which may then affect the SIgA levels in milk.^6^, ^13^, ^49^, ^50^ However, to our knowledge, this is the first study to report correlations between SIgA and HMOs, and the underlying mechanisms require more in‐depth exploration.

This study has several strengths, such as using a quantitative method of analysis (HPAEC‐PAD), collecting samples at two distinct stages of lactation (colostrum and mature milk), and focusing on the most highly expressed HMOs. However, there are also certain limitations that need to be acknowledged. The lack of previous studies investigating our specific outcomes made it challenging to accurately estimate the expected effect size. Consequently, a priori power analysis was not conducted, which may have affected the determination of an optimal sample size. Instead, we aimed to include a sufficient sample size considered commonly accepted for most statistical analyses. However, a risk of type II error still exists, where smaller true differences could remain undetected, particularly regarding the effect of the supplementation on the HMO levels and diversity. There is also the risk of unmeasured confounding variables influencing our findings. Although it is infeasible to account for all potential confounders, we investigated several demographic characteristics that did not differ statistically between the supplementation groups, except for maternal allergy, which was lower in the OP group. To address this imbalance, we performed multivariate analyses and found no interaction between supplementation groups and maternal allergy. Otherwise, randomization should have minimized the effect of other unmeasured confounders. Another potential limitation is the lack of specific data on compliance with the milk sample collection procedures and adherence to supplementation intake in this study. Nonetheless, enrolled women were required to actively participate in the study through frequent prenatal and postnatal visits for checkups, sample collection, receiving supplement bottles and returning empty ones, completing surveys, and infant follow‐ups until 2 years of age. This continuous engagement of families with a history of allergy in this allergy prevention trial may reflect a reasonable degree of adherence to the study protocols and guidelines.

In conclusion, we showed that maternal supplementation with ω‐3 PUFA decreases the HMO diversity over time. While several studies have previously investigated differences in milk components between allergic and non‐allergic mothers, this is the first study to report differences in HMO concentrations. We demonstrated that several HMOs were significantly lower in milk from allergic mothers compared to non‐allergic mothers. Finally, we identified several correlations between HMOs and SIgA in milk, indicating a possible interaction between the two mediators. This interaction may occur locally within the mammary gland or be mediated through the maternal gut microbiota and the gut‐breast axis.

## AUTHOR CONTRIBUTIONS

**Ahmed Al‐Kaabawi:** Conceptualization; data curation; formal analysis; methodology; writing – original draft; writing – review and editing; investigation; visualization; validation. **Eva Landberg:** Conceptualization; data curation; formal analysis; methodology; investigation; writing – review and editing; visualization; validation. **Magalí Martí:** Data curation; formal analysis; methodology; software; visualization; writing – review and editing; validation. **Elisabet Severin:** Methodology; investigation. **Lina Tingö:** Conceptualization; investigation; methodology; writing – review and editing. **Karel Duchén:** Methodology; investigation; resources; project administration; writing – review and editing. **Maria C. Jenmalm:** Conceptualization; funding acquisition; project administration; methodology; resources; supervision; writing – review and editing; validation.

## FUNDING INFORMATION

This study was financially supported by grants from the Swedish Research Council (2019–00989 and 2022–00595), the Swedish Heart and Lung Foundation (20200301), the Joanna Cocozza Foundation for Pediatric Research (2020–01041 and 2022–00506), the Cancer and Allergy Foundation, and the Faculty of Medicine and Health Sciences at Linköping University.

## CONFLICT OF INTEREST STATEMENT

MC Jenmalm received funding for a clinical trial and honoraria for lectures from BioGaia AB and received consultant fees and travel support from Danone Nutricia and Abigo Medical. The other authors declare no conflict of interest.

## Supporting information


Figure S1.

Appendix S1.

Table S1.
