# Supplementary material for: Effects of maternal allergy and supplementation with ω‐3 fatty acid and probiotic on human milk oligosaccharides
Source: Pediatr Allergy Immunol. 2025 Aug 1;36(8):e70162. doi: 10.1111/pai.70162 (PMC12314855; doi:10.1111/pai.70162)
Supplement: Supplementary file 1 — Figure S1. [file PAI-36-e70162-s003.docx]

Supplementary figures

**Figure S1.** Bar graphs showing the median concentrations and percentages of HMOs in colostrum and mature milk collected 3 months postpartum. Overall, HMO levels are significantly higher in colostrum than in mature milk. 3-FL is the only HMO that increases over time, while LDFT, LNFP II, and LST b remain stable, and all other HMOs decline. Fucosylated HMOs were the predominant type, followed by the neutral and sialylated HMOs in both colostrum and mature milk.

**
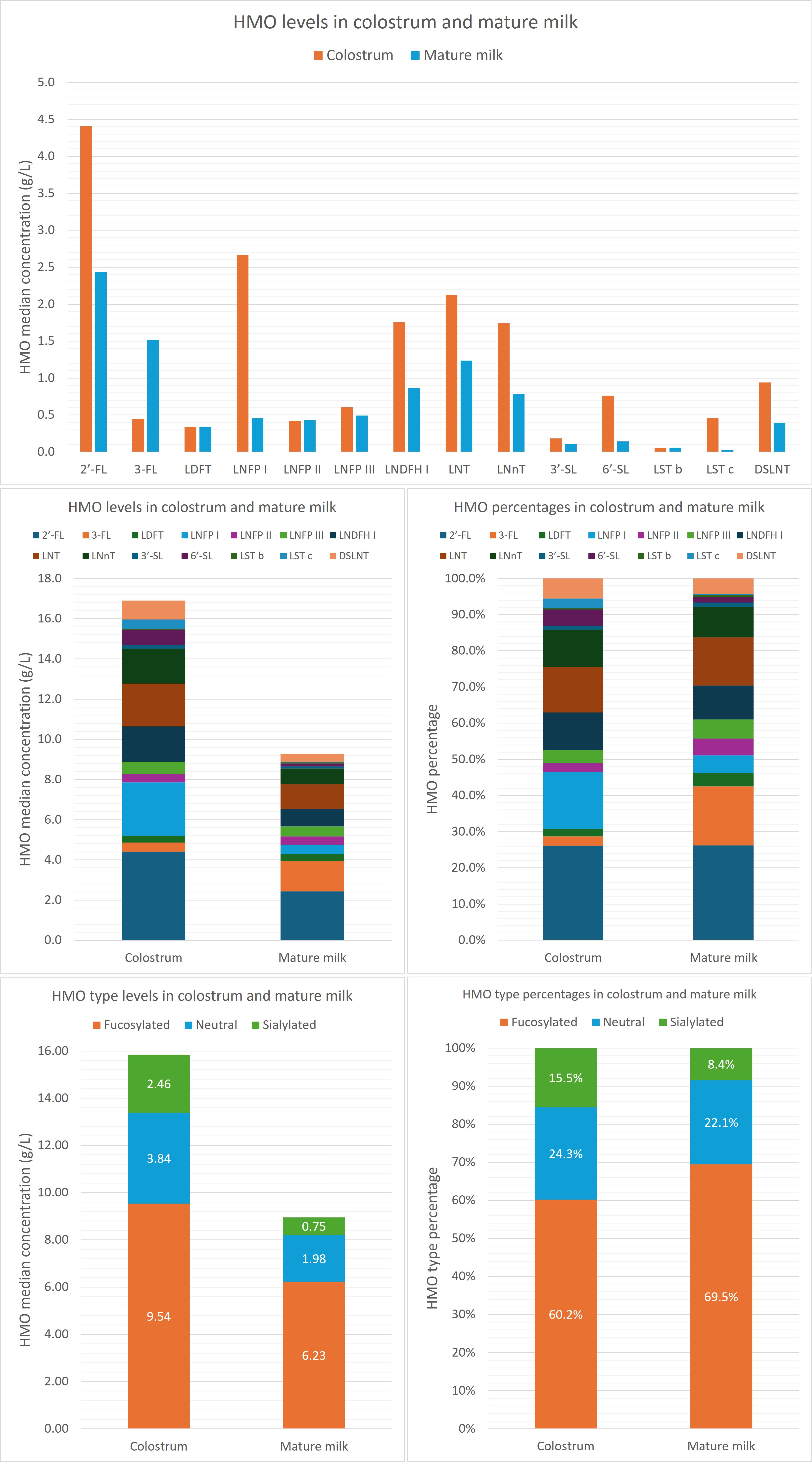
**

**Figure S2.** Boxplots with median and whiskers extending from minimum to maximum showing the HMO diversity in colostrum and mature milk across the four milk phenotypes. The diversity was the highest in the Se (Se^+^Le^+^, n=95) milk at both time points. The HMO diversity in the nSe (Se^-^Le^+^, n=25) was the second highest in colostrum and the third in mature milk. The low number of samples in the Se^+^Le^-^ (n=13) and Se^-^Le^-^ (n=3) phenotypes may not be accurately representative of the general population.

**
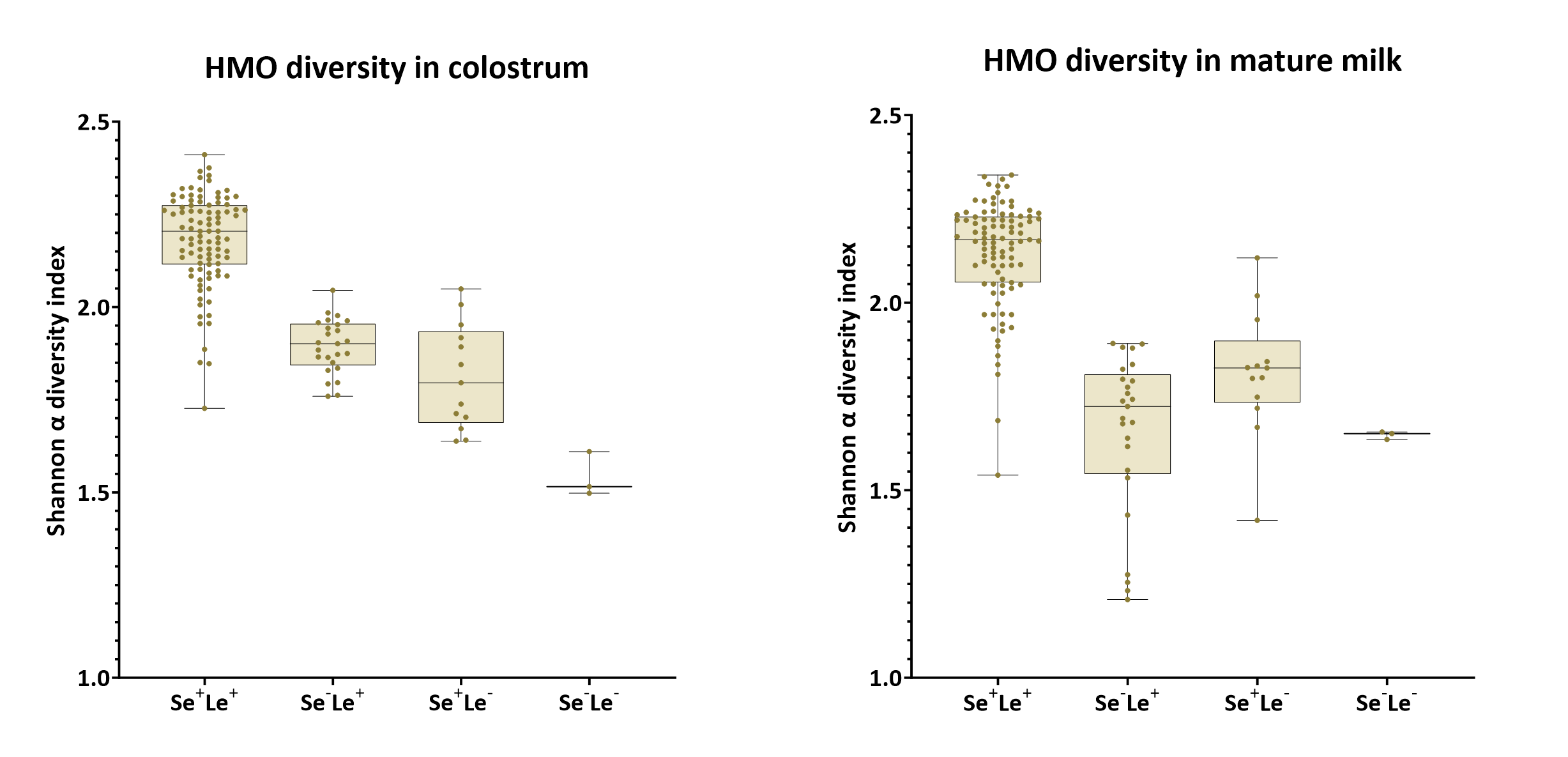
**

**Figure S3.** Boxplots with median and whiskers extending from minimum to maximum demonstrate the significant differences in HMO levels between non-allergic (n=53) and allergic mothers (n=83) in mature milk. The adjusted p-value (q) was calculated using the Kruskal-Wallis test, followed by Benjamini-Hochberg correction at a 5% FDR. A q-value below 0.05 was considered statistically significant.

**
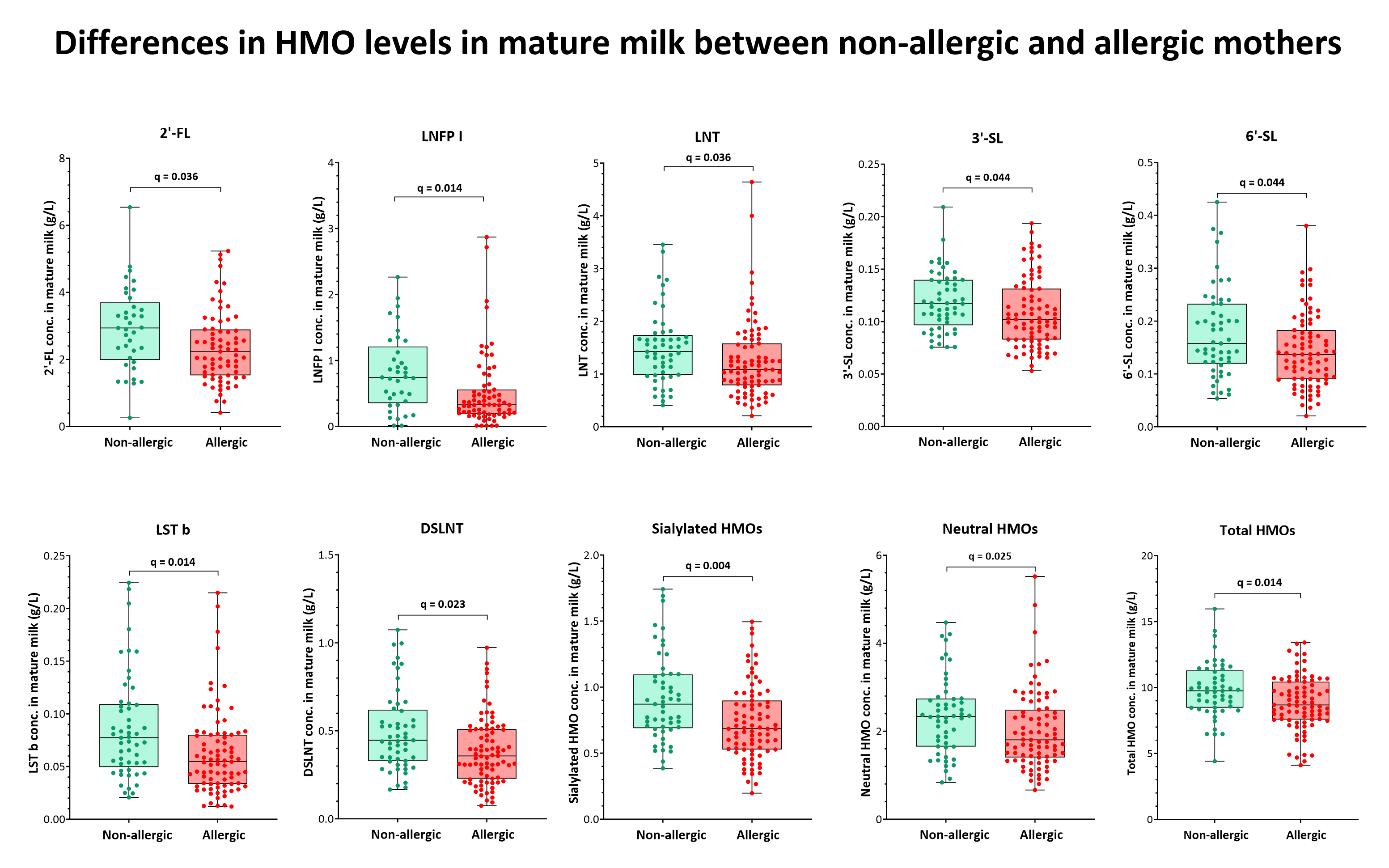
**

**Figure S4.** Heatmaps illustrate the direction and magnitude of association between HMOs in colostrum and 3-month milk and SIgA in colostrum, 1-, 2-, 3-, and 4-month milk. SIgA shows weak to moderate positive correlations with fucosylated and subsequently total HMOs, a weak negative correlation with sialylated HMOs, and no significant correlation with neutral HMOs. LDFT and LNDFH I demonstrate the strongest association with SIgA at different time points. ******correspond to p<0.001.

**
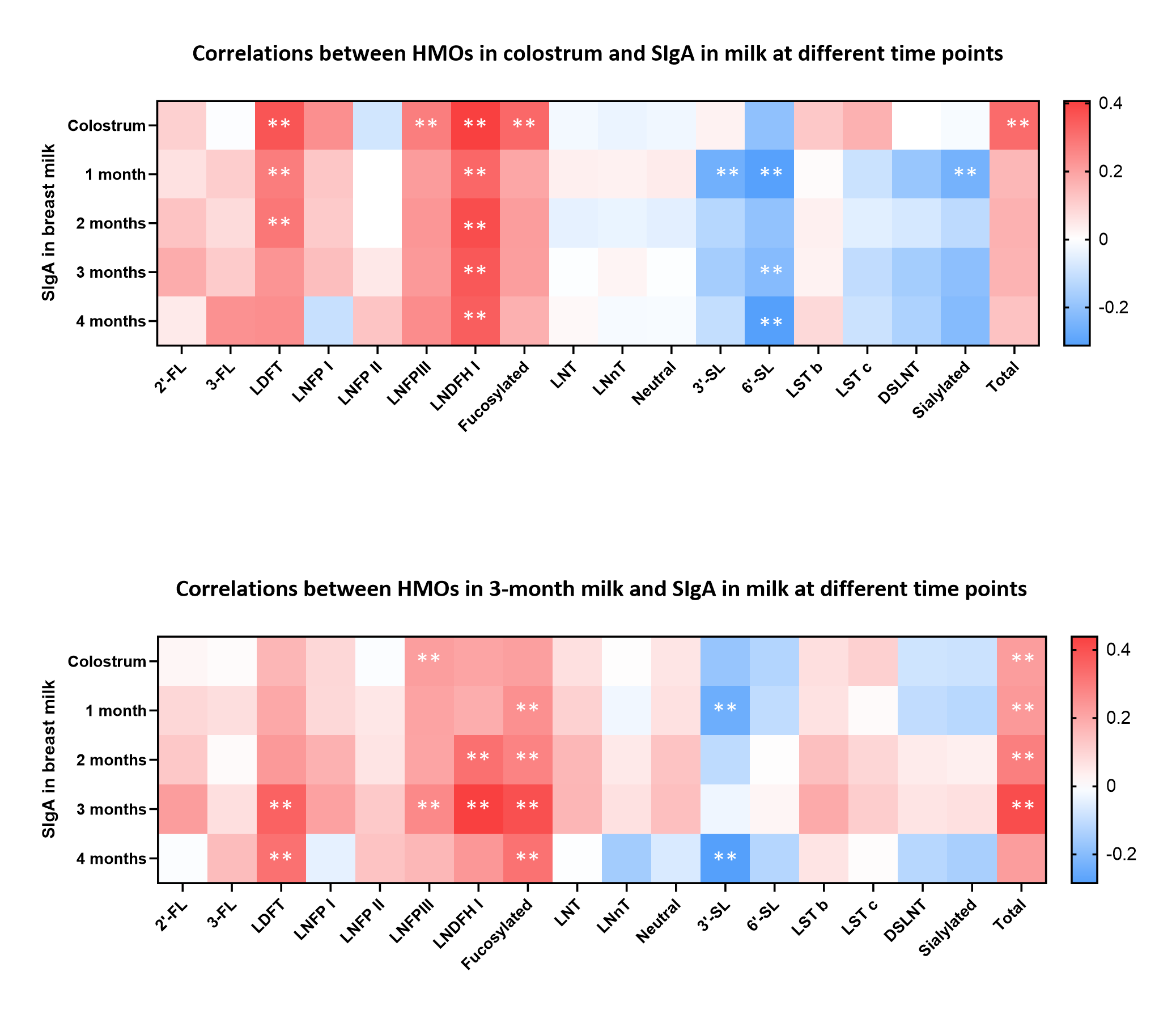
**

**Figure S5.** Scatter plots show the association between SIgA and fucosylated and total HMOs, in corresponding Se colostrum and 3-month milk. A) In Se colostrum (n=94), SIgA correlated positively with fucosylated (r_s_=0.33, p=0.0011) and total HMOs (rs=0.30, p=0.30). B) In Se 3-month milk (n=93), SIgA correlated positively with fucosylated (r_s_=0.48, p<0.0001) and total HMOs (r_s_=0.42, p<0.0001). A simple linear regression line (solid line) is fitted through the data.

**
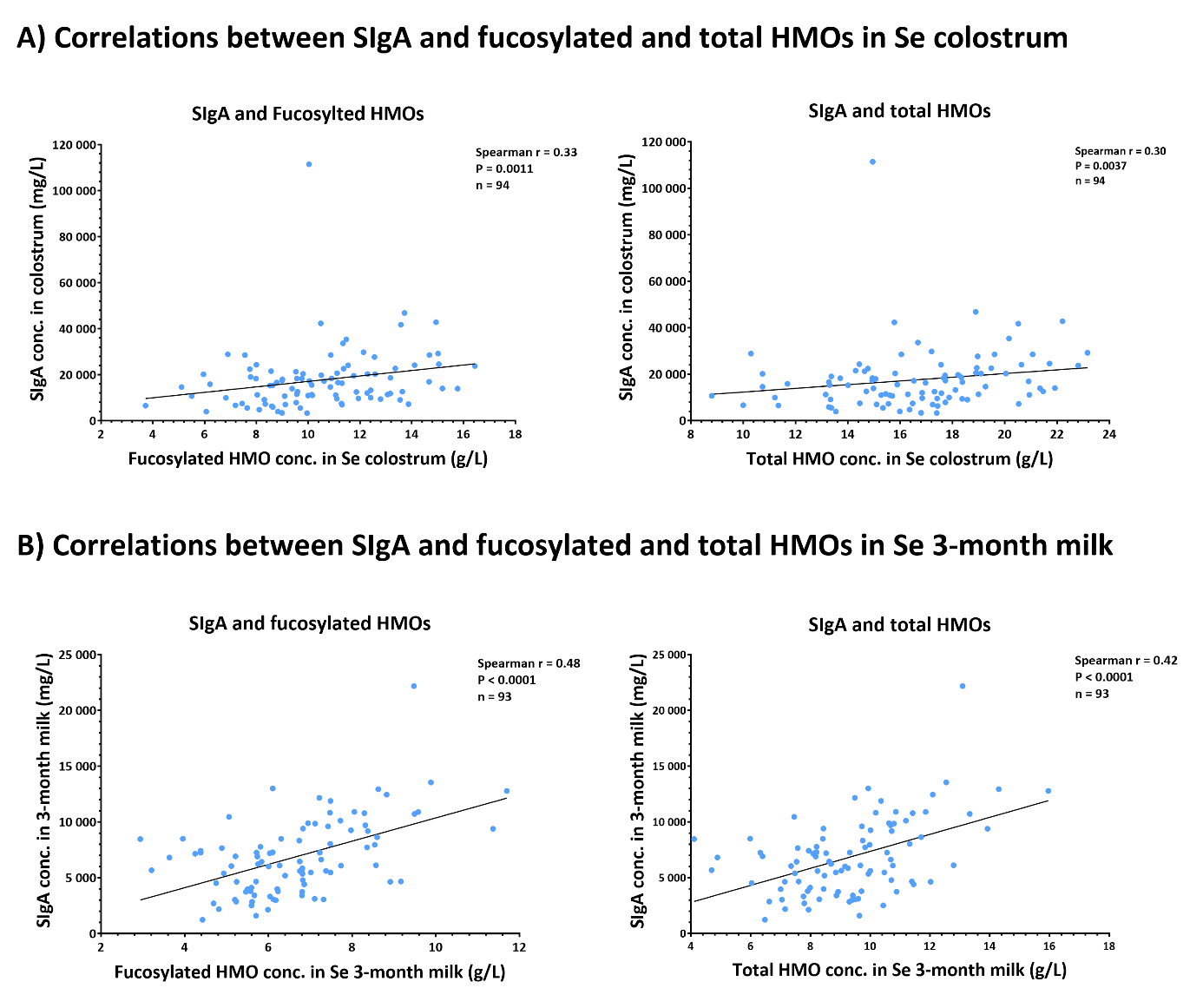
**
