# Supplementary material for: Effects of maternal allergy and supplementation with ω‐3 fatty acid and probiotic on human milk oligosaccharides
Source: Pediatr Allergy Immunol. 2025 Aug 1;36(8):e70162. doi: 10.1111/pai.70162 (PMC12314855; doi:10.1111/pai.70162)
Supplement: Supplementary file 2 — Appendix S1. [file PAI-36-e70162-s002.docx]

Supplementary file 1: detailed information on experimental procedures

HMO purification

Milk samples from 136 women were collected 1-3 days after delivery (colostrum) and three months postpartum (mature milk) and stored in a -70 °C freezer. On the day of purification, the milk sample was thawed at room temperature (RT) and a 1 mL aliquot was transferred to a 2 mL microtube (Eppendorf AG, Hamburg, Germany). To remove the milk fat, the aliquot was centrifuged at 20,000 g for 15 minutes at RT, and 0.75 mL of the infranatant was transferred to a new microtube, while carefully avoiding the fat layer on top and the cell pellet at the bottom. Again, the skimmed milk aliquot was centrifuged at 20,000 g for another 15 minutes at RT and 0.5 mL of the infranatant was transferred to a new microtube. After that, 50 µl of an internal standard consisting of 2.4 mg/mL galacturonic acid (Sigma-Aldrich, St Louis, MO, USA) and 12 mg/mL stachyose (Sigma-Aldrich) was added to each sample. To remove milk proteins, 1 mL of refrigerated 99.5% ethanol was added to the sample and kept in the fridge at 4 °C for 1 hour. Subsequently, the mixture was centrifuged at 20,000 g for 10 min at 4 °C, and 1 mL of the supernatant was transferred to a new microtube while carefully avoiding the protein precipitate. To remove hydrophobic substances, the 1 mL sample was eluted through an ISOLUTE C18 column (Biotage, Uppsala, Sweden), preconditioned with 2 mL 95% methanol followed by 2 mL of ultrapure Milli-Q water. The eluate was collected and ultra-filtrated using Amicon Ultra-4 (Merck Millipore, Cork, Ireland) with a 10 kDa molecular weight cut-off. The Amicon Ultra-4 tube was centrifuged at 4000 g for 40 minutes at RT, and the filtered sample was collected. To remove ethanol from the sample, the tube was placed on a heating block, set at 40 °C, under a sample concentrator using airflow for 30-45 minutes. At this point, the sample is ready for sialylated HMO analysis and only needed dilution of 1:100 for colostrum and 1:40 for 3-month milk samples with Milli-Q water. For neutral oligosaccharides, the sample was further purified using an anion exchange bonded silica cartridge (LC-SAX, Supelco, Bellefonte, PA, USA), preconditioned with 2 mL 95% methanol followed by 2 mL of ultrapure Milli-Q water. The LC-SAX column was used to remove acidic interferents as well as acidic HMOs from the sample and prevent the coelution of neutral and acidic HMOs during analysis. After conditioning, 50 µL of the sample was applied to the LC-SAX column, followed by 500 µL of Milli-Q. The eluate was collected and further diluted 1:50 for both time points using Milli-Q water.

HMO analysis

High-performance anion-exchange chromatography with pulsed amperometric detection (HPAEC-PAD) was used to separate and quantify 14 major HMOs in the Human milk samples (Table 1). The HPAEC-PAD system (ICS-3000, Dionex, Sunnyvale, CA, USA) was equipped with a gradient pump, a CarboPac PA-200 column (3 × 50 mm guard column and 3 × 250 mm analytical column), an electrochemical gold (Au) detector, an Ag/AgCl reference electrode, and an autosampler (AS3500). Three eluants were used in the analysis, namely 0.2 M NaOH, 0.1 M NaOH/0.5 M NaOAc, and Milli-Q water. All eluents were filtered and degassed before use and constantly held under helium pressure. HMO separation was achieved using three different gradient programs (Table S1) starting with a 20 μL sample injection volume. All sialylated HMOs were analysed with program 1, except LST c which was analysed with program 2. Both programs 1 and 2 had the same flow rate of 0.5 mL/minute, but program 1 was run at a temperature of 30 °C while program 2 was run at 40 °C to achieve optimal separation of LST c from 6’-SL. Programs 1 and 2 had a constant concentration of 100 mM NaOH and a two-step gradient of NaOAc from 20 mM to 80 mM at 5 to 30 minutes and from 80 mM to 200 mM at 30 to 40 minutes. For neutral oligosaccharides, program 3 was used with a flow rate of 0.4 mL/minute and temperature set at 25 °C. This program had a constant concentration of 20 mM NaOH and a gradient with NaOAc from 0 to 25 mM at 6 to 37 minutes. A short washing step with 100 mM NaOH/0.5 M NaOAc was included in all programs followed by 5 minutes of equilibration in preparation for the next sample. The different HMOs were identified by comparing their retention times to those of known milk oligosaccharide standards. All oligosaccharide standards were bought from Dextra Laboratories (Reading, UK), except for DSLNT, 3’-SL, and 6’-SL, which were from Sigma-Aldrich. The oligosaccharide concentrations were calculated using the individual HMOs' peak areas in relation to the area of the corresponding standard and the internal standard (galacturonic acid for sialylated and stachyose for neutral oligosaccharides).

Detection limits were determined based on peak size and concentration. Peaks in the HPAEC chromatogram were considered detectable if they met both criteria: an area greater than 0.01 nC*min and a height exceeding 0.05 nC. These thresholds corresponded to 10 times the signal-to-noise ratio. For sialylated HMOs, the detection limit was set at 3 mg/L, except for DSLNT, which had a higher limit of 20 mg/L. For neutral HMOs, the detection limit was 25 mg/L. Concentrations below these thresholds were assigned a value of half the detection limit. Secretor status was determined by the presence of detectable 2′-FL, while Lewis status was assessed based on the presence of detectable LNFP II. Missing HMOs due to Secretor or Lewis status were given no value. In this paper, Secretor Lewis positive (Se^+^Le^+^) are referred to as Se, while non-Secretor Lewis positive (Se^-^Le^+^) as nSe. HMO types, including fucosylated, neutral, and sialylated, as well as total HMOs, were calculated as the sum of the corresponding measured HMOs. Fucosylated HMOs were calculated as the combined levels of 2’-FL, 3-FL, LDFT, LNFP I, LNFP II, LNFP III, and LNDFH I. Sialylated HMOs represented the sum of 3’-SL, 6’-SL, LST b, LST c, and DSLNT. Neutral HMOs comprised the sum of LNT and LNnT, while total HMOs were calculated as the sum of the 14 measured HMOs.

The samples were analysed in 18 batches, ensuring that each participant's colostrum and mature milk samples were included in the same batch to minimise inter-batch variability. A control 3-month milk sample (Se^-^Le^+^) was incorporated into each batch to assess inter-batch variability. The mean coefficient of variation (C.V.%) for the 10 detectable HMOs in the Se-Le+ control sample was 20.2%, with no individual HMO exceeding a C.V.% of 30% (Table S2).

Secretory IgA in breast milk

Breast milk samples were collected from 136 mothers at 1-3 days after delivery (colostrum), and at 1 month, 2 months, 3 months, and 4 months postpartum, and stored in a -70 °C freezer. On the day of the analysis, the milk samples were thawed at RT and centrifuged at 680 g for 10 minutes, to remove fat and cells, and the supernatants were transferred to new tubes. The skimmed milk went through another round of centrifugation at 10000 g for 30 minutes to remove the remaining fat. In-house Enzyme-Linked Immunosorbent Assay (ELISA) was used to measure the Secretory IgA (SIgA) levels in milk as follows. A half area Costar 3690 plate (Corning, Kennebunk, ME, USA) was coated with monoclonal anti-human secretory component antibody (IgA, GA-1, Sigma Aldrich) diluted 1:10000 in PBS. The plate was first incubated in a humid chamber at 37 °C for 2 hours and then moved to a fridge and incubated at 4 °C for 3 more hours. After incubations, the plate was washed 4 times with a PBS-Tween using an automated washer (Tecan Hydroflex, Tecan Austria GmbH, Grödig, Austria). After washing, the plate was blocked with PBS + 0.5% BSA (Sigma Aldrich), and incubated for 2 hours at 37°C in a humid chamber, followed by an overnight incubation at 4°C. The next day, a seven-point standard curve (Human IgA, I1010, Sigma Aldrich) was prepared in steps of 1:2 dilution (8000-125 ng/ml) in PBS-T + 0.5% BSA (Sigma Aldrich). The plate was washed, and the standard curve was added to the plate along with diluted milk samples, a control sample, and a blank (PBS-T + 0.5% BSA) in duplicates, and was incubated at 37 °C in a humid chamber for 1 hour. The plate was washed again and an anti-human peroxidase-conjugated detection antibody (A0295, Sigma Aldrich), diluted 1:25000 in PBS-T + 0.5% BSA, was added and incubated for 1 hour in a humid chamber at 37 °C. After washing, a 3,3′,5,5′-Tetramethylbenzidine (TMB, Sigma Aldrich) substrate was added to the plate and incubated for 30 minutes at RT. The enzymatic reaction was stopped using 1.8 M H_2_SO_4_ (Scharlab, Barcelona, Spain), and the plate was read in an ELISA reader (SpectraMax ABS plus, Molecular Devices, San Jose, CA, USA) at 405 nm (reference wavelength 540 nm). The SIgA level was the average reading of the duplicates, and all duplicates had a C.V.% below 15%. The Inter-batch variability was 23.2%.
