# Supplementary material for: Effects of maternal allergy and supplementation with ω‐3 fatty acid and probiotic on human milk oligosaccharides
Source: Pediatr Allergy Immunol. 2025 Aug 1;36(8):e70162. doi: 10.1111/pai.70162 (PMC12314855; doi:10.1111/pai.70162)
Supplement: Supplementary file 3 — Table S1. [file PAI-36-e70162-s001.docx]

Supplementary tables

**Table S1.** Different HPAEC gradient programs used in HMO analysis. Programs 1 and 2 were used for sialylated HMO analysis, while program 3 was used to analyse the fucosylated and neutral HMOs.

| **Program** | **Time (min)** | **200 mM NaOH (%)** | **100 mM NaOH/0.5 M NaOAc (%)** | **H_2_O (%)** |
| --- | --- | --- | --- | --- |
| 1 and 2 | 0 | 48 | 4 | 48 |
|  | 5 | 48 | 4 | 48 |
|  | 30 | 42 | 16 | 42 |
|  | 40 | 30 | 40 | 30 |
|  | 43 | 0 | 100 | 0 |
|  | 47 | 0 | 100 | 0 |
|  | 50 | 48 | 4 | 48 |
|  | 55 | 48 | 4 | 48 |
| 3 | 0 | 10 | 0 | 90 |
|  | 6 | 10 | 0 | 90 |
|  | 37 | 7.5 | 5 | 87.5 |
|  | 44 | 0 | 100 | 0 |
|  | 46 | 0 | 100 | 0 |
|  | 50 | 10 | 0 | 90 |
|  | 55 | 10 | 0 | 90 |

**Table S2.** HMO levels (g/L) and C.V.% values for the Se^-^Le^+^ control 3-month milk sample included in each of the 18 batches of sample analysis. All HMOs showed a C.V.% below 30%, with an average C.V.% of 20.2% for the 10 measured HMOs.

| **Batches** | **3-FL** | **LNFP II** | **LNFP III** | **LNT** | **LNnT** | **3'-SL** | **6'-SL** | **LST b** | **LST c** | **DSLNT** | **Fucosylated** | **Neutral** | **Sialylated** | **Total** |
| --- | --- | --- | --- | --- | --- | --- | --- | --- | --- | --- | --- | --- | --- | --- |
| Batch 1 | 3.75 | 0.55 | 0.23 | 0.48 | 0.50 | 0.09 | 0.13 | 0.04 | 0.01 | 0.29 | 4.53 | 0.98 | 0.56 | 6.07 |
| Batch 2 | 3.53 | 0.53 | 0.22 | 0.45 | 0.45 | 0.09 | 0.13 | 0.04 | 0.01 | 0.28 | 4.29 | 0.89 | 0.55 | 5.73 |
| Batch 3 | 3.84 | 0.57 | 0.30 | 0.64 | 0.57 | 0.08 | 0.13 | 0.04 | 0.01 | 0.22 | 4.70 | 1.21 | 0.48 | 6.39 |
| Batch 4 | 3.74 | 0.57 | 0.24 | 0.65 | 0.53 | 0.08 | 0.14 | 0.05 | 0.01 | 0.25 | 4.55 | 1.19 | 0.53 | 6.27 |
| Batch 5 | 3.52 | 0.74 | 0.35 | 0.54 | 0.44 | 0.06 | 0.09 | 0.04 | 0.01 | 0.17 | 4.62 | 0.97 | 0.37 | 5.96 |
| Batch 6 | 3.71 | 0.82 | 0.24 | 0.61 | 0.41 | 0.08 | 0.12 | 0.05 | 0.01 | 0.35 | 4.78 | 1.03 | 0.62 | 6.42 |
| Batch 7 | 3.64 | 0.83 | 0.27 | 0.69 | 0.46 | 0.08 | 0.12 | 0.05 | 0.01 | 0.30 | 4.73 | 1.16 | 0.56 | 6.45 |
| Batch 8 | 3.43 | 0.70 | 0.23 | 0.61 | 0.41 | 0.07 | 0.11 | 0.04 | 0.01 | 0.26 | 4.36 | 1.02 | 0.50 | 5.88 |
| Batch 9 | 4.25 | 0.86 | 0.26 | 0.77 | 0.30 | 0.08 | 0.13 | 0.06 | 0.02 | 0.34 | 5.37 | 1.08 | 0.62 | 7.07 |
| Batch 10 | 3.61 | 0.71 | 0.19 | 0.66 | 0.41 | 0.08 | 0.11 | 0.04 | 0.01 | 0.27 | 4.51 | 1.08 | 0.51 | 6.10 |
| Batch 11 | 5.78 | 1.07 | 0.33 | 0.76 | 0.55 | 0.09 | 0.14 | 0.06 | 0.01 | 0.36 | 7.18 | 1.31 | 0.66 | 9.16 |
| Batch 12 | 5.73 | 0.99 | 0.29 | 0.71 | 0.50 | 0.08 | 0.13 | 0.05 | 0.01 | 0.27 | 7.01 | 1.21 | 0.55 | 8.77 |
| Batch 13 | 5.00 | 0.79 | 0.47 | 0.65 | 0.55 | 0.06 | 0.10 | 0.04 | 0.01 | 0.20 | 6.26 | 1.19 | 0.41 | 7.86 |
| Batch 14 | 3.97 | 1.03 | 0.28 | 0.71 | 0.62 | 0.05 | 0.07 | 0.03 | 0.01 | 0.18 | 5.28 | 1.33 | 0.33 | 6.94 |
| Batch 15 | 5.67 | 0.98 | 0.28 | 0.74 | 0.50 | 0.07 | 0.11 | 0.05 | 0.01 | 0.25 | 6.93 | 1.24 | 0.50 | 8.67 |
| Batch 16 | 5.69 | 0.99 | 0.32 | 0.92 | 0.63 | 0.06 | 0.11 | 0.07 | 0.02 | 0.25 | 7.01 | 1.56 | 0.51 | 9.08 |
| Batch 17 | 4.22 | 0.80 | 0.49 | 0.65 | 0.49 | 0.05 | 0.08 | 0.06 | 0.02 | 0.17 | 5.51 | 1.14 | 0.37 | 7.03 |
| Batch 18 | 4.08 | 0.79 | 0.47 | 0.68 | 0.49 | 0.07 | 0.11 | 0.07 | 0.01 | 0.21 | 5.35 | 1.18 | 0.48 | 7.00 |
| **C.V.%** | **20.17** | **21.46** | **29.70** | **16.63** | **16.46** | **16.79** | **16.83** | **21.40** | **19.36** | **23.13** | **19.09** | **13.53** | **17.80** | **16.42** |

**Table S3.** The distribution of maternal demographic characteristics and milk phenotypes between non-allergic and allergic mothers. The table shows the median and standard deviation of continuous variables, including age, start of supplementation, and time of delivery, and count and percentages for number of participants, mode of delivery, baby’s sex, exclusive breastfeeding at 3 months, and milk phenotypes. There were no significant differences in demographic characteristics or milk phenotypes between non-allergic and allergic mothers. Two non-allergic mothers had missing data for age, start of supplementation, and time of delivery. One non-allergic mother had missing data for the mode of delivery and the baby’s sex. Seven non-allergic mothers and six allergic mothers had missing data for exclusive breastfeeding at 3 months.

|  | **Non-allergic** | **Allergic** | **Total** |
| --- | --- | --- | --- |
| **Characteristics** |  |  |  |
| Number of participants | 53 (39%) | 83 (61%) | 136 (100%) |
| Age (years) | 31 (4.6) | 31 (4) | 31 (4.2) |
| Start of supplementation (weeks) | 20 (1.9) | 20 (1.6) | 20 (1.7) |
| Time of delivery (weeks) | 40 (1.7) | 40 (1.6) | 40 (1.6) |
| Mode of delivery (Caesarean) | 6 (11.5%) | 5 (6%) | 11 (8.1%) |
| Baby’s sex (male) | 24 (46.2%) | 37 (44.5%) | 61 (44.9%) |
| Exclusive breastfeeding at 3 months | 34 (73.9%) | 60 (77.9%) | 94 (69.1%) |
| **Milk phenotypes** |  |  |  |
| Se^+^Le^+^ | 31 (58.5%) | 64 (77.1%) | 95 (70%) |
| Se^-^Le^+^ | 14 (26.4%) | 11 (13.3%) | 25 (18%) |
| Se^+^Le^-^ | 6 (11.3%) | 7 (8.4%) | 13 (10%) |
| Se^-^Le^-^ | 2 (3.8%) | 1 (1.2%) | 3 (2%) |

**Table S4.** Number of missing data points from the mother demographic characteristics in each of the four supplementation groups. Supplementation groups: OL (ω-3 PUFA + *L. reuteri*), OP (ω-3 PUFA + Placebo), PL (Placebo + *L. reuteri*), and PP (Placebo + Placebo).

| **Characteristics** | OL | OP | PL | PP | Total |
| --- | --- | --- | --- | --- | --- |
| Number of participants | 36 | 30 | 36 | 34 | 136 |
| Age (years) | 0 | 0 | 0 | 1 | 1 |
| Start of supplementation (weeks) | 0 | 0 | 0 | 1 | 1 |
| Time of delivery (weeks) | 0 | 0 | 1 | 1 | 2 |
| Mode of delivery (Caesarean) | 0 | 0 | 0 | 1 | 1 |
| Baby’s sex (male) | 0 | 0 | 0 | 1 | 1 |
| Exclusive breastfeeding at 3 months | 4 | 1 | 5 | 3 | 13 |
| Maternal allergy (allergic) | 0 | 0 | 0 | 0 | 0 |

**Table S5.** This table shows the results of the Aligned Ranked Transform (ART) ANOVA investigating the interaction effect between the HMO diversity change over time, from colostrum to mature milk, and maternal allergy (non-allergic vs allergic) within each supplementation group (OL, OP, PL, PP). This nonparametric approach was used because the data did not meet the assumptions of the two-way repeated measures ANOVA. Similar to the Wilcoxon matched-pairs signed-rank test, the results show that HMO diversity changed significantly over time. Also, the maternal allergy status does not affect the HMO diversity change over time, and most importantly, there is no interaction between the diversity change over time and maternal allergy status across all supplementation groups. Supplementation groups: OL (ω-3 PUFA + *L. reuteri*), OP (ω-3 PUFA + Placebo), PL (Placebo + *L. reuteri*), and PP (Placebo + Placebo). p<0.001=**, p<0.0001=***. Df: degree of freedom, Df.res: degree of freedom of the residuals. Pr(>F): p-value for ART ANOVA.

| **Group** | **Effect** | **F** | **Df** | **Df.res** | **Pr(>F)** |
| --- | --- | --- | --- | --- | --- |
| OL | Diversity change | 15.47614 | 1 | 34 | **0.00039 ***** |
|  | Maternal allergy | 3.05523 | 1 | 34 | 0.08950 |
|  | Diversity change* Maternal allergy | 0.49021 | 1 | 34 | 0.48859 |
| OP | Diversity change | 9.700008 | 1 | 28 | **0.00422 **** |
|  | Maternal allergy | 0.073434 | 1 | 28 | 0.78838 |
|  | Diversity change* Maternal allergy | 0.049933 | 1 | 28 | 0.82480 |
| PL | Diversity change | 3.83155 | 1 | 34 | 0.05854 |
|  | Maternal allergy | 0.76290 | 1 | 34 | 0.38855 |
|  | Diversity change* Maternal allergy | 0.07504 | 1 | 34 | 0.78579 |
| PP | Diversity change | 2.63248 | 1 | 32 | 0.11451 |
|  | Maternal allergy | 0.99069 | 1 | 32 | 0.32704 |
|  | Diversity change* Maternal allergy | 0.53786 | 1 | 32 | 0.46866 |

**Table S6.** Significant differences in HMO concentrations between non-allergic and allergic mothers. In colostrum, non-allergic mothers (n=53) had higher 2’-FL, LNFP I, and LST b levels and lower HMO diversity than allergic mothers (n=83). However, these differences in colostrum did not survive the FDR. In mature milk (3 months), several HMO concentrations were significantly higher in non-allergic mothers (n=53) compared to allergic ones (n=83). In the Se subpopulation, non-allergic mothers (n=31) had significantly higher LNFP I, sialylated, fucosylated, and total HMOs than allergic ones (n=64). Mann–Whitney *U* test was used for group comparisons, and a p-value <0.05 was considered significant. Significant differences that did not pass the FDR at 5% were marked in red. Adjusted p-values (q) were calculated with Benjamini-Hochberg's method at 5% FDR. Maternal allergy was self-reported and subsequently verified by a clinician based on reported symptoms and included eczema, asthma, food allergy, or allergic rhinoconjunctivitis.

| Colostrum | | | Mature milk | | |
| --- | --- | --- | --- | --- | --- |
| HMO | **P value** | **Adjusted p-value (q)** | **HMO** | **P value** | **Adjusted p-value (q)** |
| 2’-FL | 0.025 | 0.117 | 2’-FL | 0.013 | 0.036 |
| LNFP I | 0.007 | 0.091 | LNFP I | 0.002 | 0.014 |
| LST b | 0.013 | 0.091 | LNT | 0.011 | 0.036 |
| Diversity | 0.026 | 0.052 | 3’-SL | 0.022 | 0.044 |
|  |  |  | 6’-SL | 0.022 | 0.044 |
|  |  |  | LST b | 0.002 | 0.014 |
|  |  |  | DSLNT | 0.005 | 0.023 |
|  |  |  | Sialylated | 0.001 | 0.004 |
|  |  |  | Neutral | 0.019 | 0.025 |
|  |  |  | Total | 0.007 | 0.014 |
| Se colostrum | | | **Se Mature milk** | | |
| HMO | **P value** | **Adjusted p-value (q)** | **HMO** | **P value** | **Adjusted p-value (q)** |
| 2’-FL | 0.015 | 0.105 | 2’-FL | 0.028 | 0.09 |
| LNFP I | 0.003 | 0.042 | LNFP I | 0.003 | 0.042 |
| DSLNT | 0.05 | 0.233 | 3’-SL | 0.017 | 0.079 |
| Sialylated | 0.035 | 0.047 | 6’-SL | 0.032 | 0.09 |
| Fucosylated | 0.019 | 0.038 | DSLNT | 0.016 | 0.079 |
| Total | 0.009 | 0.036 | Sialylated | 0.003 | 0.006 |
|  |  |  | Fucosylated | 0.008 | 0.011 |
|  |  |  | Total | 0.002 | 0.006 |

**Table S7.** This table shows the results of the two-way permutational multivariate analysis of variance (PERMANOVA) investigating the interaction effect between maternal allergy and supplementation. This test was used because our data violates the multivariate ANOVA (MANOVA) assumptions. Two-way PERMANOVA was performed on the significantly different HMOs between non-allergic and allergic mothers. **A)** LNFP I, sialylated, fucosylated, and total HMOs in Se^+^Le^+^ colostrum. **B)** LNFP I, sialylated, fucosylated, and total HMOs in Se^+^Le^+^ mature milk. **C)** LNT, 3’-SL, 6’-SL, LST b, DSLNT, sialylated, neutral, and total HMOs in mature milk. Two HMOs, 2’-FL and LNFP I, were excluded from this multivariate analysis because they are produced only by secretor mothers (Se^+^Le^+^ and Se^+^Le^-^) and are missing for non-secretors. Including them would have excluded non-secretor mothers from the PERMANOVA analysis. Therefore, they have been analysed separately. **D)** 2’-FL and LNFP I in mature milk. There was no significant interaction between supplementation and maternal allergy in any of the multivariate analyses. In these two-way PERMANOVA tests, we used a permutational N of 9999 and Euclidean distance as a similarity index. Homogeneity tests of multivariate dispersions (PERMDISP) indicated no significant difference in dispersion among groups, supporting the assumption of equal group variances required for PERMANOVA. Significance codes: p<0.01=*, p<0.001=**, p<0.0001=****, Df: degree of freedom, Pr(>F): p-value from permutation test.

**A. HMOs in Se+Le+ colostrum**

| **Source** | **Sum of squares** | **Df** | **Pseudo-R^2^** | **F** | **Pr(>F)** |
| --- | --- | --- | --- | --- | --- |
| Supplementation | 35.05 | 3 | 0.02042 | 0.6670 | 0.5992 |
| Maternal allergy | 115.69 | 1 | 0.06740 | 6.6036 | **0.0087 **** |
| Interaction | 41.46 | 3 | 0.02416 | 0.7889 | 0.5282 |
| Residual | 1524.18 | 87 | 0.88802 |  |  |
| Total | 1716.38 | 94 | 1.00000 |  |  |

**B. HMOs in Se+Le+ mature milk**

| **Source** | **Sum of squares** | **Df** | **Pseudo-R^2^** | **F** | **Pr(>F)** |
| --- | --- | --- | --- | --- | --- |
| Supplementation | 35.22 | 3 | 0.05127 | 1.9094 | 0.1298 |
| Maternal allergy | 72.49 | 1 | 0.10552 | 11.790 | **0.0009 ***** |
| Interaction | 44.35 | 3 | 0.06456 | 2.4043 | 0.0677 |
| Residual | 534.91 | 87 | 0.77866 |  |  |
| Total | 686.97 | 94 | 1.00000 |  |  |

**C. HMOs in mature milk**

| **Source** | **Sum of squares** | **Df** | **Pseudo-R^2^** | **F** | **Pr(>F)** |
| --- | --- | --- | --- | --- | --- |
| Supplementation | 6.41 | 3 | 0.00801 | 0.3733 | 0.8566 |
| Maternal allergy | 39.19 | 1 | 0.04893 | 6.8432 | **0.0044 **** |
| Interaction | 22.27 | 3 | 0.02781 | 1.2963 | 0.2567 |
| Residual | 733.12 | 128 | 0.91525 |  |  |
| Total | 801.00 | 135 | 1.00000 |  |  |

**D. 2’-FL and LNFP I in mature milk**

| **Source** | **Sum of squares** | **Df** | **Pseudo-R^2^** | **F** | **Pr(>F)** |
| --- | --- | --- | --- | --- | --- |
| Supplementation | 1.898 | 3 | 0.01049 | 0.3909 | 0.8251 |
| Maternal allergy | 9.739 | 1 | 0.05384 | 6.0189 | **0.0100 *** |
| Interaction | 7.436 | 3 | 0.04111 | 1.5318 | 0.1914 |
| Residual | 161.810 | 100 | 0.89456 |  |  |
| Total | 180.883 | 107 | 1.00000 |  |  |

**Table S8.** These tables show the nonparametric correlation (Spearman’s rho) between HMOs in colostrum and 3-month milk and SIgA in colostrum, 1-, 2-, 3-, and 4-month milk. Tables A and B show the correlation in all samples, Tables C and D show the correlation in secretors (Se) milk, and Tables E and F show the correlation in non-secretors (nSe) milk. Only HMOs with significant correlations were shown. * Corresponds to p-value <0.05, and **corresponds to p-value <0.01. N: number of samples. Sig: significance level.

**A. Correlations between HMOs in colostrum and SIgA in colostrum, 1-, 2-, 3-, and 4-month milk**

| **SIgA** | **3-FL** | **LDFT** | **LNFP I** | **LNFP III** | **LNDFH I** | **3’-SL** | **6’-SL** | **Sialylated** | **Fucosylated** | **Total** |
| --- | --- | --- | --- | --- | --- | --- | --- | --- | --- | --- |
| **Colostrum** | -0.007 | **0.360**** | 0.237* | **0.274**** | **0.406**** | 0.027 | -0.206* | -0.015 | **0.321**** | **0.315**** |
| Sig. (2-tailed) | 0.934 | **0** | 0.014 | **0.001** | **0** | 0.756 | 0.017 | 0.865 | **0** | **0** |
| N | 135 | **107** | 107 | **135** | **94** | 135 | 135 | 135 | **135** | **135** |
| **1-month milk** | 0.106 | **0.276**** | 0.12 | 0.209* | **0.323**** | **-0.261**** | **-0.310**** | **-0.254**** | 0.187* | 0.152 |
| Sig. (2-tailed) | 0.237 | **0.005** | 0.233 | 0.018 | **0.002** | **0.003** | **0** | **0.004** | 0.035 | 0.089 |
| N | 127 | **100** | 100 | 127 | **89** | **127** | **127** | **127** | 127 | 127 |
| **2-month milk** | 0.076 | **0.287**** | 0.112 | 0.223* | **0.378**** | -0.135 | -0.201* | -0.122 | 0.208* | 0.165 |
| Sig. (2-tailed) | 0.393 | **0.003** | 0.265 | 0.011 | **0** | 0.128 | 0.022 | 0.168 | 0.018 | 0.061 |
| N | 129 | **102** | 102 | 129 | **90** | 129 | 129 | 129 | 129 | 129 |
| **3-month milk** | 0.11 | 0.227* | 0.137 | 0.215* | **0.355**** | -0.166 | **-0.224**** | -0.211* | 0.205* | 0.16 |
| Sig. (2-tailed) | 0.205 | 0.02 | 0.163 | 0.013 | **0** | 0.057 | **0.01** | 0.015 | 0.018 | 0.066 |
| N | 133 | 105 | 105 | 133 | **93** | 133 | **133** | 133 | 133 | 133 |
| **4-month milk** | 0.234* | 0.240* | -0.103 | 0.245* | **0.341**** | -0.111 | **-0.312**** | -0.225* | 0.166 | 0.13 |
| Sig. (2-tailed) | 0.027 | 0.043 | 0.392 | 0.02 | **0.007** | 0.298 | **0.003** | 0.033 | 0.117 | 0.224 |
| N | 90 | 71 | 71 | 90 | **62** | 90 | **90** | 90 | 90 | 90 |

**B. Correlations between HMOs in 3-month milk and SIgA in colostrum, 1-, 2-, 3-, and 4-month milk**

| **SIgA** | **LDFT** | **LNFP I** | **LNFP III** | **LNDFH I** | **3’-SL** | **6’-SL** | **LST b** | **Fucosylated** | **Total** |
| --- | --- | --- | --- | --- | --- | --- | --- | --- | --- |
| **Colostrum** | 0.167 | 0.091 | **0.221**** | 0.208* | -0.174* | -0.129 | 0.072 | 0.220* | **0.223**** |
| Sig. (2-tailed) | 0.085 | 0.351 | **0.01** | 0.044 | 0.043 | 0.137 | 0.409 | 0.01 | **0.009** |
| N | 107 | 107 | **135** | 94 | 135 | 135 | 135 | 135 | **135** |
| **1-month milk** | 0.199* | 0.088 | 0.210* | 0.187 | **-0.245**** | -0.107 | 0.067 | **0.254**** | **0.233**** |
| Sig. (2-tailed) | 0.047 | 0.381 | 0.018 | 0.079 | **0.005** | 0.233 | 0.457 | **0.004** | **0.008** |
| N | 100 | 100 | 127 | 89 | **127** | 127 | 127 | **127** | **127** |
| **2-month milk** | 0.234* | 0.178 | 0.208* | **0.327**** | -0.109 | 0.004 | 0.146 | **0.283**** | **0.294**** |
| Sig. (2-tailed) | 0.018 | 0.074 | 0.018 | **0.002** | 0.217 | 0.964 | 0.098 | **0.001** | **0.001** |
| N | 102 | 102 | 129 | **90** | 129 | 129 | 129 | **129** | **129** |
| **3-month milk** | **0.360**** | 0.216* | **0.269**** | **0.439**** | -0.028 | 0.022 | 0.195* | **0.400**** | **0.408**** |
| Sig. (2-tailed) | **0** | 0.027 | **0.002** | **0** | 0.746 | 0.801 | 0.024 | **0** | **0** |
| N | **105** | 105 | **133** | **93** | 133 | 133 | 133 | **133** | **133** |
| **4-month milk** | **0.326**** | -0.043 | 0.165 | 0.238 | **-0.284**** | -0.124 | 0.061 | **0.323**** | 0.224* |
| Sig. (2-tailed) | **0.006** | 0.722 | 0.12 | 0.062 | **0.007** | 0.245 | 0.57 | **0.002** | 0.034 |
| N | **71** | 71 | 90 | 62 | **90** | 90 | 90 | **90** | 90 |

**C. Correlations between HMOs in Se colostrum and SIgA in colostrum, 1-, 2-, 3-, and 4-month milk**

| **SIgA** | **3-FL** | **LDFT** | **LNFP III** | **LNDFH I** | **3’-SL** | **6’-SL** | **LST b** | **Sialylated** | **Fucosylated** | **Total** |
| --- | --- | --- | --- | --- | --- | --- | --- | --- | --- | --- |
| **Colostrum** | 0.177 | **0.396**** | **0.289**** | **0.406**** | -0.042 | -0.152 | 0.227* | 0.05 | **0.331**** | **0.297**** |
| Sig. (2-tailed) | 0.089 | **0** | **0.005** | **0** | 0.689 | 0.143 | 0.028 | 0.631 | **0.001** | **0.004** |
| N | 94 | **94** | **94** | **94** | 94 | 94 | 94 | 94 | **94** | **94** |
| **1-month milk** | 0.237* | **0.291**** | 0.17 | **0.323**** | **-0.287**** | **-0.289**** | 0.064 | -0.210* | 0.204 | 0.146 |
| Sig. (2-tailed) | 0.025 | **0.006** | 0.112 | **0.002** | **0.006** | **0.006** | 0.552 | 0.048 | 0.055 | 0.172 |
| N | 89 | **89** | 89 | **89** | **89** | **89** | 89 | 89 | 89 | 89 |
| **2-month milk** | 0.213* | **0.337**** | 0.189 | **0.378**** | -0.118 | -0.103 | 0.074 | -0.003 | 0.261* | 0.216* |
| Sig. (2-tailed) | 0.044 | **0.001** | 0.074 | **0** | 0.267 | 0.332 | 0.487 | 0.975 | 0.013 | 0.041 |
| N | 90 | **90** | 90 | **90** | 90 | 90 | 90 | 90 | 90 | 90 |
| **3-month milk** | 0.254* | **0.269**** | 0.162 | **0.355**** | -0.192 | -0.171 | 0.071 | -0.15 | **0.291**** | 0.222* |
| Sig. (2-tailed) | 0.014 | **0.009** | 0.122 | **0** | 0.065 | 0.101 | 0.499 | 0.152 | **0.005** | 0.032 |
| N | 93 | **93** | 93 | **93** | 93 | 93 | 93 | 93 | **93** | 93 |
| **4-month milk** | **0.479**** | 0.270* | 0.205 | **0.341**** | -0.234 | **-0.331**** | 0.144 | -0.252* | 0.201 | 0.083 |
| Sig. (2-tailed) | **0** | 0.034 | 0.109 | **0.007** | 0.067 | **0.009** | 0.265 | 0.048 | 0.118 | 0.52 |
| N | **62** | 62 | 62 | **62** | 62 | **62** | 62 | 62 | 62 | 62 |

**D. Correlations between HMOs in Se 3-month milk and SIgA in colostrum, 1-, 2-, 3-, and 4-month milk**

| **SIgA** | **3-FL** | **LDFT** | **LNFP III** | **LNDFH I** | **3’-SL** | **LST b** | **Fucosylated** | **Total** |
| --- | --- | --- | --- | --- | --- | --- | --- | --- |
| **Colostrum** | 0.193 | 0.207* | 0.207* | 0.208* | -0.118 | 0.112 | 0.238* | 0.196 |
| Sig. (2-tailed) | 0.063 | 0.045 | 0.046 | 0.044 | 0.258 | 0.282 | 0.021 | 0.059 |
| N | 94 | 94 | 94 | 94 | 94 | 94 | 94 | 94 |
| **1-month milk** | 0.156 | 0.209* | 0.083 | 0.187 | -0.212* | 0.067 | 0.244* | 0.184 |
| Sig. (2-tailed) | 0.143 | 0.049 | 0.442 | 0.079 | 0.046 | 0.534 | 0.021 | 0.085 |
| N | 89 | 89 | 89 | 89 | 89 | 89 | 89 | 89 |
| **2-month milk** | 0.113 | **0.283**** | 0.103 | **0.327**** | -0.051 | 0.175 | **0.309**** | 0.264* |
| Sig. (2-tailed) | 0.288 | **0.007** | 0.334 | **0.002** | 0.63 | 0.098 | **0.003** | 0.012 |
| N | 90 | **90** | 90 | **90** | 90 | 90 | **90** | 90 |
| **3-month milk** | 0.192 | **0.433**** | 0.188 | **0.439**** | 0.009 | 0.232* | **0.481**** | **0.422**** |
| Sig. (2-tailed) | 0.066 | **0** | 0.071 | **0** | 0.93 | 0.025 | **0** | **0** |
| N | 93 | **93** | 93 | **93** | 93 | 93 | **93** | **93** |
| **4-month milk** | **0.330**** | 0.321* | 0.091 | 0.238 | -0.274* | 0.044 | 0.303* | 0.193 |
| Sig. (2-tailed) | **0.009** | 0.011 | 0.483 | 0.062 | 0.031 | 0.737 | 0.017 | 0.134 |
| N | **62** | 62 | 62 | 62 | 62 | 62 | 62 | 62 |

**E. Correlations between HMOs in nSe colostrum and SIgA in colostrum, 1-, 2-, 3-, and 4-month milk**

| **SIgA** | **LNFP III** | **6’-SL** |
| --- | --- | --- |
| **Colostrum** | **0.576**** | -0.261 |
| Sig. (2-tailed) | **0.003** | 0.208 |
| N | **25** | 25 |
| **1-month milk** | **0.643**** | -0.399 |
| Sig. (2-tailed) | **0.001** | 0.053 |
| N | **24** | 24 |
| **2-month milk** | **0.615**** | -0.480* |
| Sig. (2-tailed) | **0.001** | 0.018 |
| N | **24** | 24 |
| **3-month milk** | **0.583**** | -0.467* |
| Sig. (2-tailed) | **0.002** | 0.019 |
| N | **25** | 25 |
| **4-month milk** | 0.562* | -0.294 |
| Sig. (2-tailed) | 0.024 | 0.269 |
| N | 16 | 16 |

**F. Correlations between HMOs in nSe 3-month milk and SIgA in colostrum, 1-, 2-, 3-, and 4-month milk**

| **SIgA** | **LNFP II** | **LNFP III** | **LNT** | **3’-SL** | **LST b** | **Fucosylated** | **Total** |
| --- | --- | --- | --- | --- | --- | --- | --- |
| **Colostrum** | 0.182 | 0.181 | 0.274 | -0.412* | 0.127 | 0.05 | 0.156 |
| Sig. (2-tailed) | 0.383 | 0.387 | 0.185 | 0.041 | 0.545 | 0.812 | 0.456 |
| N | 25 | 25 | 25 | 25 | 25 | 25 | 25 |
| **1-month milk** | **0.574**** | 0.470* | 0.33 | -0.429* | 0.36 | 0.359 | **0.573**** |
| Sig. (2-tailed) | **0.003** | 0.021 | 0.116 | 0.037 | 0.084 | 0.085 | **0.003** |
| N | **24** | 24 | 24 | 24 | 24 | 24 | **24** |
| **2-month milk** | 0.515* | 0.272 | 0.458* | -0.327 | 0.442* | 0.18 | 0.497* |
| Sig. (2-tailed) | 0.01 | 0.198 | 0.024 | 0.119 | 0.031 | 0.4 | 0.014 |
| N | 24 | 24 | 24 | 24 | 24 | 24 | 24 |
| **3-month milk** | **0.618**** | 0.333 | 0.298 | -0.282 | 0.347 | 0.434* | **0.652**** |
| Sig. (2-tailed) | **0.001** | 0.104 | 0.147 | 0.173 | 0.089 | 0.03 | **0** |
| N | **25** | 25 | 25 | 25 | 25 | 25 | **25** |
| **4-month milk** | 0.474 | 0.25 | 0.165 | -0.579* | 0.271 | 0.397 | 0.515* |
| Sig. (2-tailed) | 0.064 | 0.35 | 0.542 | 0.019 | 0.311 | 0.128 | 0.041 |
| N | 16 | 16 | 16 | 16 | 16 | 16 | 16 |
